# Mechanism Analysis of Acid Tolerance Response of *Bifidobacterium longum* subsp. *longum* BBMN 68 by Gene Expression Profile Using RNA-Sequencing

**DOI:** 10.1371/journal.pone.0050777

**Published:** 2012-12-07

**Authors:** Junhua Jin, Bing Zhang, Huiyuan Guo, Jianyun Cui, Lu Jiang, Shuhui Song, Min Sun, Fazheng Ren

**Affiliations:** 1 Key Laboratory of Functional Dairy, College of Food Science and Nutritional Engineering, China Agricultural University, Beijing, China; 2 Core Genomic Facility, Beijing Institute of Genomics, Chinese Academy of Sciences, Beijing, China; 3 Beijing Higher Institution Engineering Research Center of Animal Product, Beijing, China; 4 Beijing Key Laboratory of Nutrition, Health and Food Safety, Beijing, China; Cornell University, United States of America

## Abstract

To analyze the mechanism of the acid tolerance response (ATR) in *Bifidobacterium longum* subsp. *longum* BBMN68, we optimized the acid-adaptation condition to stimulate ATR effectively and analyzed the change of gene expression profile after acid-adaptation using high-throughput RNA-Seq. After acid-adaptation at pH 4.5 for 2 hours, the survival rate of BBMN68 at lethal pH 3.5 for 120 min was increased by 70 fold and the expression of 293 genes were upregulated by more than 2 fold, and 245 genes were downregulated by more than 2 fold. Gene expression profiling of ATR in BBMN68 suggested that, when the bacteria faced acid stress, the cells strengthened the integrity of cell wall and changed the permeability of membrane to keep the H^+^ from entering. Once the H^+^ entered the cytoplasm, the cells showed four main responses: First, the F_0_F_1_-ATPase system was initiated to discharge H^+^. Second, the ability to produce NH_3_ by cysteine-cystathionine-cycle was strengthened to neutralize excess H^+^. Third, the cells started NER-UVR and NER-VSR systems to minimize the damage to DNA and upregulated HtpX, IbpA, and γ-glutamylcysteine production to protect proteins against damage. Fourth, the cells initiated global response signals ((p)ppGpp, polyP, and Sec-SRP) to bring the whole cell into a state of response to the stress. The cells also secreted the quorum sensing signal (AI-2) to communicate between intraspecies cells by the cellular signal system, such as two-component systems, to improve the overall survival rate. Besides, the cells varied the pathways of producing energy by shifting to BCAA metabolism and enhanced the ability to utilize sugar to supply sufficient energy for the operation of the mechanism mentioned above. Based on these reults, it was inferred that, during industrial applications, the acid resistance of bifidobacteria could be improved by adding BCAA, γ-glutamylcysteine, cysteine, and cystathionine into the acid-stress environment.

## Introduction

Several species in the genus *Bifidobacterium* are considered probiotic, and their presence has been associated with healthy microbiota [1]. This has led to their widespread application in probiotic products. During storage, distribution and delivery, bifidobacteria are exposed to acid stress due to the production of organic acid in fermented foods. In addition, during ingestion, bacteria must survive passage through the low-pH environment of the stomach to reach their ecological niche. Acid stress is a major challenge to bifidobacteria. It can cost them their viability and reduce their probiotic effects [2], [3]. Strengthening acid tolerance is critical to survival of *Bifidobacterium* and therefor of impact on the quality and functionality of the probiotic product.

Pre-exposure to sublethal pH has been shown to increase the viability of several bacteria during subsequent exposure to the lethal pH. The phenomenon is known as the acid tolerance response (ATR). There are many studies of ATR in microorganisms, such as *Streptococcus mutans* [4], [5], *Lactococcus lacti*s [6], [7], [8], *Bifidobacterium longum*, and *Bifidobacterium animalis* [9], [10], [11].

Our previous study suggested that the genes involved in ATR in bifidobacteria differed from those of other bacteria [12]. However, there have been very few studies on the mechanism behind ATR in bifidobacteria. Only Waddington et al. and Sanchez et al. have investigated ATR of *Bifidobacterium longum*. They did so by comparing the 2D-gel protein profile [13], [14]. They demonstrated that the response of the *B. longum* to acid stress involved the maintenance of pH homeostasis by H^+^- ATPase and the production of NH_3_. However, mechanisms underlying acid tolerance used by Gram-positive bacteria are complex. Several mechanisms of ATR in lactic acid bacteria (LAB) have been proposed, including the maintenance of pH homeostasis [5], [15], protecting DNA and protein [16], [17], [18], neutralizing the excessive H^+^ [19], [20] as well as signal communication [5], [21], [22]. For advising to improve the acid resistance of bifidobacteria in industrial applications, it is necessary to decipher whether and how these mechanisms involve ATR.

In order to determine how bifidobacteria combat acid stress, *Bifidobacterium longum* subsp. *longum* BBMN68, a novel isolate of a centenarian, was used as a model strain. Previous studies have indicated that BBMN68 was a potential probiotic strain. *B. longum* subsp. *longum* BBMN68 demonstrated to improve intestinal function and enhance immunity in healthy mice [23], [24]. The complete genome sequence of BBMN68 is available at NCBI database (NC_014656.1) [25]. In this study, we investigated the conditions to stimulate the ATR in BBMN68 and its genome-wide gene expression by high-throughput RNA-Seq using the platforms of the Genome Analyzer of Applied Biosystems (SOLiD). Ultimately, this study illustrated the gene expression patterns underlying ATR in *B. longum* subsp. *longum* in detail. These results may inspire new strategies toward enhancing the industrial utility of this species.

## Materials and Methods

### Selection of optimal acid-adaptation conditions

*Bifidobacterium longum* subsp. *longum* BBMN68 (CGMCC 2265, China General Microbiological Culture Collection Center) was cultured in modified MRS medium (supplemented with 0.05% L-cysteine) at an initial pH of 6.5 at 37°C, under anaerobic conditions.

To select the challenge pH of BBMN68, the impact of pH on survival was tested. A part of the exponential cells (OD_600_ = 0.6) were collected. The collected cells were resuspended in isovolumetric modified MRS adjusted to pH 5.0, 4.5, 4.2, 4.0, and 3.5 with HCl and then incubated at 37°C for 120 min under anaerobic conditions. The other part of the cells (OD_600_ = 0.6) served as control cells. The survival cultures were counted by plate count on modified MRS agar medium. The pH at which the survival ratio of the cells decreased to 1% of control cells was defined as challenge pH.

To select the adaptation pH, the impact of pre-incubation at different pH on the survival ratio at the challenge pH was tested. A portion of the exponential cells (OD_600_ = 0.6) served as control cells. The remaining exponential cells were collected and resuspended in isovolumetric modified MRS adjusted to pH 5.0, 4.5, 4.2, and 4.0 and allowed to pre-incubate at 37°C. After 120 min the control and pre-incubated cells were harvested and resuspended in modified MRS adjusted to pH 3.5 for acid challenge. The acid-challenged cells were incubated at 37°C for 30, 60, 90, and 120 min. Serial dilutions of the cells incubated for different periods of time were spread on modified MRS agar medium. The survival rate was calculated by dividing the number of cfu per ml after incubation at pH 3.5 by the number of cfu per ml of the control cells.

### RNA sequencing

Total RNA was isolated from the acid-adapted cells (pre-incubated at pH 4.5 for 120 min before challenge at pH 3.5) and the control cells using Trizol. Total RNA was purified using a Ribo-minus Kit (Invitrogen, Cat. no. K1083708, U.S.) to deplete ribosomal RNA. The RNA-Seq library was constructed according to manufacturer's protocol from SOLiD™. Total RNA-Seq Kit, starting from 1 µg rRNA-depleted RNA. Briefly, the following components were added to the mixture on ice, in order: 8 µl RNA (1 µg), 1 µl 10× RNase III buffer, and 1 µl RNase III (Applied Biosystems, Cat. no. AM2290, U.S.). The mixture was incubated at 37°C for 10 min and at 65°C for 20 min. Then RiboMinus™ Concentration Module (Invitrogen, Cat. no. K155005, U.S.) was used to clean the fragmented rRNA and concentrate the rRNA-depleted RNA in the mixture. Selective hybridization of rRNA to the RiboMinus™ Probe and removal of rRNA using RiboMinus™ Magnetic Beads were proceeded following the instructions ot kit. cDNA was synthesized by addition of 19 µl RT Master Mix and incubated at 42°C for 30 min. The cDNA was purified using the MinElute PCR Purification Kit (Qiagen Cat. no. 28006, Germany). 150–200 bp range cDNA was selected with Novex pre-cast gel products (Invitrogen, Cat. no. NP0322BOX, U.S.). The cDNA was amplified with 15 PCR cycles and cleaned up with PureLink PCR Micro Kit (Invitrogen, Cat. no. K310250, U.S.). A cDNA library was sequenced using an AB SOLiD 4.0 sequencer according to the manufacturer's instructions.

The details of the methods for alignment of read and quantization of gene expression were represented in Peng Cui's study [26]. All sequenced reads were aligned to the *Bifidobacterium longum* subsp. *longum* BBMN68 (NC_014656.1) using AB's SOLiD Corona_lite_v4.2 software available at http://waprna.big.ac.cn. We used a recursive strategy to improve the usable sequenced reads information, that is 50 mers reads were first mapped to the genome with a tolerance of ≤5 mismatches. The reads that failed to be mapped were progressively trimmed off, with five bases at one time from the 3′ end. After this, the reads were mapped to the genome again until a match was found (unless the read had been trimmed by <30 bases). All those uniquely mapped reads were used for calculating the genes RPKM values (reads per kilobase of exon per million mapped sequenced reads), which is used for defining gene expression level. We identified differential expressed genes (DEG) from different samples according to an R package named DEGseq (http://waprna.big.ac.cn/rnaseq/function/degseq.jsp), p<0.001 [27].

### Validation of RNA-Seq data by real-time PCR

Validation of RNA-Seq data for 15 different genes was performed by real-time PCR as described by Vandecasteele [28]. The primers of selected genes were designed based on *Bifidobacterium longum* subsp. *longum* BBMN68 genome using Primer-blast software in the online NCBI blast database and synthesized by Invitrogen. The primer pairs are summarized in Table 1. The amplification efficiency of each primer set was determined using the standard curve. Each RT-PCR mixture with a total volume of 25 µl contained 9 µl of SYBR Green Real-time PCR Master Mix (Tiangen, Cat. no. FP202-02, China), 0.5 µM of the forward and reverse primers, 2 µl of cDNA template, and nuclease-free water. Real-time PCR was carried out with a Techne Quantica real-time PCR detection system (Techne, U.K.) under the following conditions: one cycle of 10 min at 95°C, 40 cycles of 15 s at 95°C and 30 s at 61°C. Determination of expression ratios was performed in duplicate for all the six biological replicates.

**Table 1:** Primers used for the verification of selected genes during RT-PCR.

Gene ID	Primer sequence (F/R: 5′-3′)	Product size (bp)	Amplification efficiency
*BBMN68_1079*	CGCAATCAGGCCACTGCCCA CGCGTCATCACACGGCCCTT	185	1.99
*BBMN68_1080*	GGGGAGCGCGATGGCAAAGT CTCCCCCGTACGCGACCAGA	210	2.00
*BBMN68_1264*	GCGTTCCTGCCGGAGCTGTT TCAGCACCGCAACGAGGCAG	190	1.96
*BBMN68_1344*	TGTTCCTCGTCAACGCGGGC GATCAGGCGCGTGGGCAACT	177	1.87
*BBMN68_626*	GTCCTACCGCGTCAAGCCCG AGTGGCCTTCAGGGACGGCA	154	2.07
*BBMN68_1635*	CCGACCCCTGGCGTGGACTA AGCTGCACCACGATGGACGC	176	1.96
*BBMN68_114*	CACACCCCGACCGAACACCG GGTGAGGGTTTCGGCCTCGC	165	1.96
*BBMN68_1694*	GCGAGGACGCCGTCATGGAG CGACCACGGGAGCCGACAAC	175	1.95
*BBMN68_270*	GTGCTGTACGCCGTGACCCC GGCGACGCAGGCGATGATGA	195	1.95
*BBMN68_1728*	GAGGTCCTGACCGGAGGCGT GTTGACTCCGGCGCGTTCCA	195	1.93
*BBMN68_195*	GTGCCGCTCGTTGGCCGTAT TGGCCTCGTCGTCCAGCAGA	235	1.97
*BBMN68_1099*	GCACATTGCAACGCGACCCC ATGGCCAGCACGGCCACATC	160	1.93
*BBMN68_1100*	CAAGCAGGCCATCCCCACCG GGCCTTGGCCACTTCCTCGG	166	1.95
*BBMN68_933*	ACTACGTGCGCCTGTTGGGC GCGCCGTACACGAAACCCCA	226	2.01
*BBMN68_1816*	GCCGTGCCACCGACATCGAA CGATGCGGGCACGATCGACA	222	1.92

### Determination of ATPase activity

To investigate the role of ATPase in ATR in BBMN68, the activity of ATPase in the control cells and the acid-adapated cells was detected. ATPase of the control cells and acid-adapted cells (incubated at pH 4.5 for 120 min) was determined based on the method described by Even et al [29]. A volume of culture corresponding to 20 mg (dry weight) of cells was centrifuged (4°C, 10 min at 6000 g), washed with MgCl_2_ (1 mM), and resuspended in MgCl_2_ (1 mM). Cells could be stored at −20°C after this step. After centrifugation (4°C, 10 min at 12,000 g), cells were resuspended in potassium phosphate buffer (73 mM, pH 6.5) containing 0.05% (v/v) Triton X-100 and incubated for 24 h at 20°C. After thawing and centrifugation (20°C, 10 min at 12,000 g), the pellet was resuspended in MgCl_2_ (1 mM) as ATPase extraction.

Determination of ATPase activity was based on ATP hydrolysis to ADP and Pi at 37°C, followed by assay of the Pi formed once the reaction was stopped. One hundred microliters of ATPase extract was added to 1.9 ml of reaction mixture containing Tris buffer (21.5 mM, pH 7.8), EDTA (0.61 mM), MgCl_2_ (5.4 mM), NaCl (105 mM) and KCl (2.4 mM). The reaction was initiated by the addition of 500 µl of ATP (20 mM, pH 7.8) and stopped by the addition of 1 ml HCl (0.2 M). Pi formed by ATP hydrolysis in the reaction mixture was measured as follows: a sample of 2 ml of the reaction mixture was incubated for 15–30 min at room temperature with 2 ml of solution containing 0.2% (w/v) ascorbic acid, 1% (w/v) ammonium heptamolybdate, and 8% (v/v) perchloric acid 70%. OD was determined at 825 nm.

### Effect of chloramphenicol treatment on acid-resistance

To investigate the relationship between the inhibition of protein synthesis and ATR in BBMN68, the acid-resistance of BBMN68 treated by chloramphenicol was assessed. One hundred micrograms per ml chloramphenicol was added into the medium containing exponential cells (OD_600_ = 0.6). The cells were incubated at 37°C for 30 min under anaerobic conditions. No effect on the survival of the cells was detected in such condition for 180 min. The control cells and the chloramphenicol-treated cells were collected and resuspended in modified MRS adjusted to the challenge pH 3.5. The cells were incubated at 37°C for up to 120 min with periodic sampling every 30 min to assess viability. The survival rate was calculated by dividing the number of cfu per ml after incubation at pH 3.5 by the number of cfu per ml of the control cells.

## Results and Discussion

### Optimal acid-adaptation conditions

**Figure 1:**
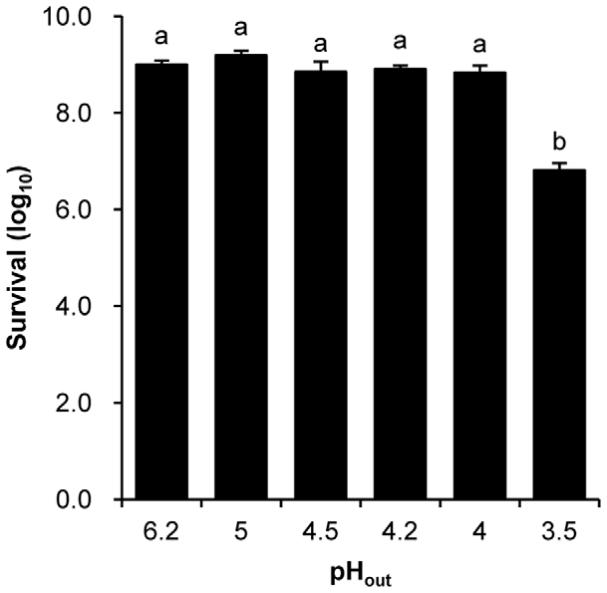
Survival of *Bifidobacterium longum* subsp. *longum* BBMN68 after 2 hours at different pH. Exponential cells were collected and resuspended into fresh MRS with different pH to culture for 2 hours under anaerobic condition. Significant differences among the groups are marked with different letters (*P*<0.05).

As shown in Figure 1, the viability of the cells treated at pH 4.0–5.0 was the same as that of control cells, while the viability of the cells treated at pH 3.5 decreased to less than 1% of control cells. Hence, pH 3.5 was chosen as the challenge pH to detect the effect of acid-adaptation.

**Figure 2:**
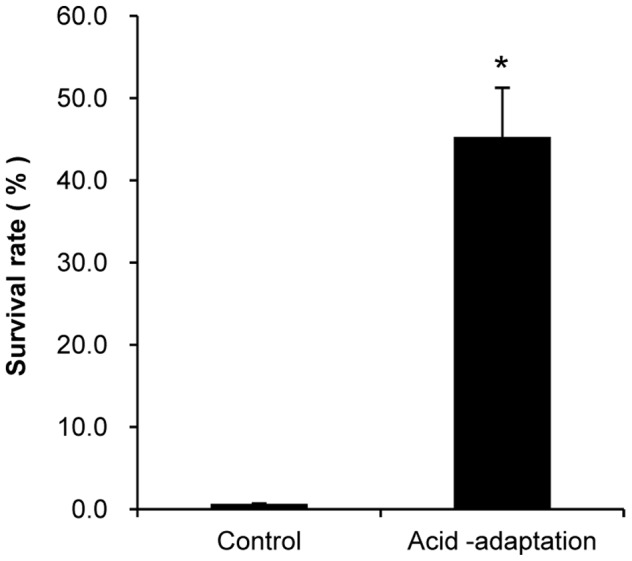
Effects of acid- adaptation on acid resistance of *Bifidobacterium longum* subsp. *longum* BBMN68. Control cells were not treated; acid-adaptation cells were acid-adapted at pH 4.5 for 2 hours; acid resistance shows the survival ratios of the cells at pH 3.5 for 2 hours. * significant difference (*P*<0.05).

To determine optimal acid-adaptation conditions, the survival rate of BBMN68 at challenge pH after pre-incubation at pH 5.0, 4.5, 4.2, and 4.0 lasting 2 hours was detected. As shown in Figure 2, the survival rate of the control cells at pH 3.5 for 120 min was 0.7%. The survival rate of the cells subjected to pre-incubation at pH 4.5 was maintained at 45% after acid challenge at pH 3.5 for 120 min. However, pre-incubation treatments at other pH levels were ineffective at protecting cells against a lethal acid challenge (data not shown). The results indicated that ATR in BBMN68 could be triggered by exposure to pH 4.5 for 120 min and therefor was chosen as the acid-adaptation treatment.

### Analysis and verification of RNA-Seq data

**Figure 3:**
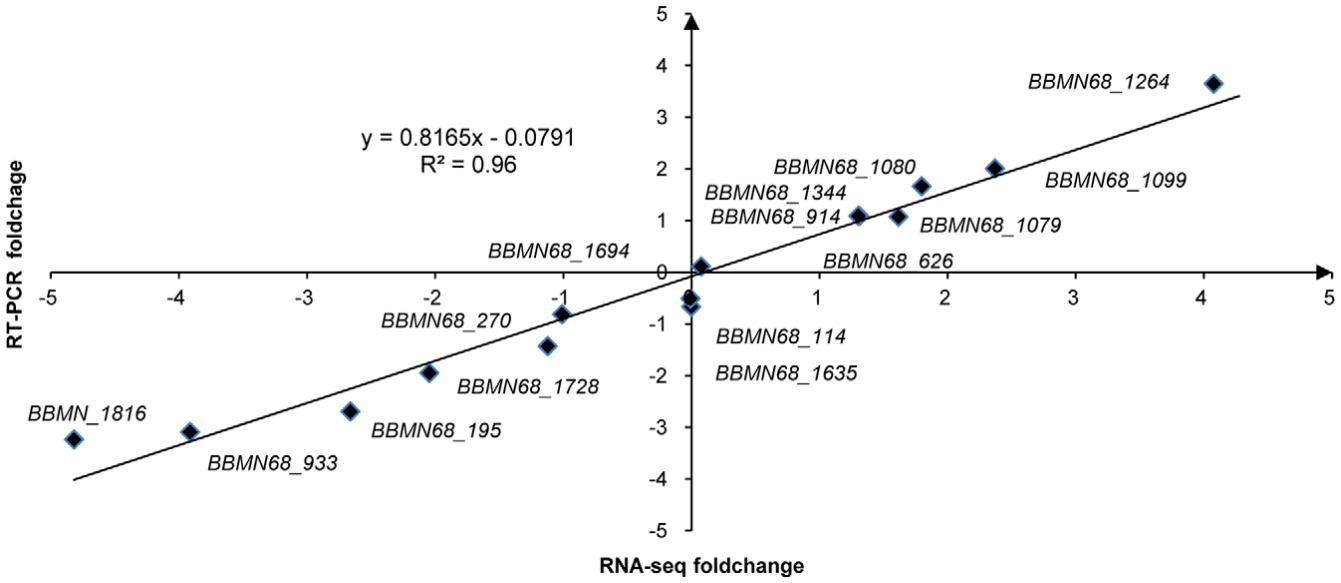
Correlation of foldchange values from RNA-Seq and RT-PCR. The R^2^ value is 0.96.

To investigate the genes involved in ATR in BBMN68, differential gene expression between the control cells and the acid-adapted cells was identified by the high-throughput RNA-Seq. The *Bifidobacterium longum* subsp. *longum* BBMN68 genome contains 1814 predicted protein-coding sequences (CDSs) and 57 tRNAs [25]. We mapped the sequenced reads to the genome. In the control cells and acid-adaptated cells, respectively, 39.3% and 44.9% of the total reads (40 million) from raw RNA-seq data were mapped uniquely to genome, while similar proportions were mapped multiplely to genome (Table S3). Only 6.4% and 3.5% of the total reads were mapped to rRNA sequence (Table S3), respectively. All those uniquely mapped reads were used for calculating the genes RPKM values to normalize the expression level of genes. The DEGseq analysis of the RNA-Seq data revealed the expression of 1480 genes (*p*<0.001) in both condition. The expression of 538 genes had changed by more than 2 fold after acid-adaptation. Of these, 293 genes were upregulated and 245 genes were downregulated. To validate the RNA-Seq data, RT-PCR of 15 genes was performed. As shown in Figure 3, there was a strong positive correlation (R^2^ = 0.96) between RNA-Seq data and RT-PCR data.

**Figure 4:**
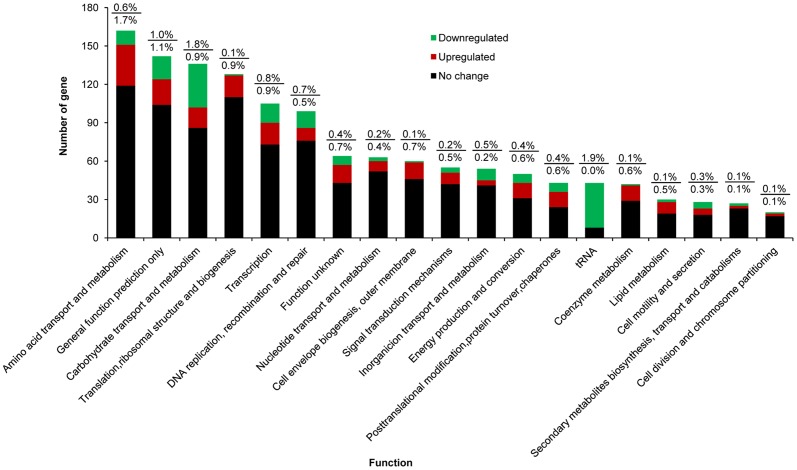
Comparsion of functions to clusters of orthologous genes in *Bifidobacterium longum* subsp. *longum* BBMN68. These genes included those whose expression was upregulated (red block), downregulated (green block), and unchanged (black block) relative to control cells. The numbers above and below the little bar indicate the enrichments of the down-regulated genes and the up-regulated genes in respect to the genome, respectively.

Of the 538 differentially expressed genes, 35 genes encoded tRNA (Figure 4). All of them were downregulated by more than 2 fold after acid-adaptation. Sequence alignment analysis indicated that 351 of 503 genes matched sequences of the cluster of orthologous genes (COGs). They were involved in 16 biological functions (Figure 4). In respect to the genome, the relative number of the DEGs involved in carbohydrate transport/metabolism (2.7%), amino acid transport/metabolism (2.3%), and Transcription (1.7%) was high. The relative number of the genes involved in secondary metabolite biosynthesis, transport, and catabolism (0.2 %), and cell division and chromosome partitioning (0.2 %) was very low. The relative numbers of genes involved in other functions ranged from 0.5 % to 1.1 %. The other 152 genes showed no COGs.

### Mechanism analysis of ATR

According to the roles of the genes in the metabolic pathway and the mechanisms of ATR listed in LABs in previous studies, the association between the DEGs and ATR in BBMN68 was mined. The results indicated that these genes involved in blocking H^+^, discharging H^+^, neutralizing H^+^, the protection of proteins (PP), repair of damaged DNA, signal transduction systems, regulation of global response and regulation of the energy supply, participated in ATR in BBMN68. Some detected genes were not reported in the ATR mechanisms in previous studies.

### Blocking H^+^

The cell envelope and membrane (CEM) are probably the first targets of physicochemical stress. They are mainly composed of lipid, peptidoglycan, and exopolysaccharides (EPS) in bifidobacteria. Fatty acid (FA) modifications of cell membrane play an important role in protection from acid stress [30], [31]. As shown in Table 2, *BBMN68_1705* encoding cyclopropane fatty acid (CFA) synthase (Cfa) was upregulated by 4.69 folds. Previous study proved that Cfa acted as an important enzyme in the acid tolerance response of bacteria through the methods of constructing *cfa*-deficient mutans and *cfa*-overexpression strains [31]. It was inferred that CFA also made important contribution to ATR in BBMN68.

**Table 2:** Changes in expression of the genes involved in biosynthesis of Cell Envelope and Membrane (CEM).

Gene ID	RPKM-control	RPKM-acid-adapted	Fold change	COG	Code	Gene	Gene description
*BBMN68_1432*	113.37	241.35	2.13*	H	COG0846		K12410, NAD-dependent deacetylase [EC:3.5.1.-]
*BBMN68_949*	140.53	283.21	2.02*	G	COG1109	*manB*	K03431, phosphoglucosamine mutase [EC:5.4.2.10]
*BBMN68_725*	77.61	174.46	2.25*	M	COG1207	*glmU*	K04042, bifunctional UDP-N-acetylglucosamine pyrophosphorylase/glucosamine-1-phosphate N-acetyltransferase [EC:2.7.7.23 2.3.1.157]
*BBMN68_956*	127.77	346.11	2.71*	EM	COG0329	*dapA*	K01714, dihydrodipicolinate synthase [EC:4.2.1.52]
*BBMN68_615*	17.64	35.36	2.00*	E	COG0624	*pePV*	K01439, succinyl-diaminopimelate desuccinylase [EC:3.5.1.18]
*BBMN68_1087*	94.27	330.69	3.51*	I	COG1211	*ispD*	K00991, 2-C-methyl-D-erythritol 4-phosphate cytidylyltransferase [EC:2.7.7.60]
*BBMN68_317*	30.69	98.5	3.21*	H	COG0142	*ispA*	K13787, geranylgeranyl diphosphate synthase, type I [EC:2.5.1.1/2.5.1.10/2.5.1.29]
*BBMN68_144*	119.96	321.03	2.68*	M	COG0821	*gcpE*	K03526, (E)-4-hydroxy-3-methylbut-2-enyl-diphosphate synthase [EC:1.17.7.1]
*BBMN68_147*	50.77	126.06	2.48*	I	COG0020	*uppS*	K00806, undecaprenyl diphosphate synthase [EC:2.5.1.31]
*BBMN68_143*	135.57	320.22	2.36*	I	COG0743	*dxr*	K00099, 1-deoxy-D-xylulose-5-phosphate reductoisomerase [EC:1.1.1.267]
*BBMN68_747*	8.28	17.69	2.14*	I	COG0245	*ispF*	K01770, 2-C-methyl-D-erythritol 2,4-cyclodiphosphate synthase [EC:4.6.1.12]
*BBMN68_1487*	390.91	785.8	2.01*	M	COG1088	*rfbB*	K01710, dTDP-glucose 4,6-dehydratase [EC:4.2.1.46]
*BBMN68_1488*	284.49	635.17	2.23*	M	COG1091	*rfbC*	K00067, dTDP-4-dehydrorhamnose reductase [EC:1.1.1.133]K01790, dTDP-4-dehydrorhamnose 3,5-epimerase [EC:5.1.3.13]
*BBMN68_1489*	666.12	861.34	1.29	M	COG1209	*rfbA*	K00973, glucose-1-phosphate thymidylyltransferase [EC:2.7.7.24]
*BBMN68_1495*	193.38	554.77	2.87*	MG	COG1682	*tagG*	K01992, ABC-2 type transport system permease protein
*BBMN68_1496*	468.32	697.82	1.49	G	COG1134	*tagH*	K01990, ABC-2 type transport system ATP-binding protein
*BBMN68_1705*	73.35	350.75	4.78*	M	COG2230	*cfa*	Cyclopropane fatty acid synthase
*BBMN68_1009*	113.58	407.27	3.59*	M	COG0463		Hypothetical protein
*BBMN68_1297*	25.47	55.92	2.20*	M	COG0463		Hypothetical protein
*BBMN68_1707*	58.19	118.96	2.04*	M	COG0463		Glycosyltransferase for cell wall membrane
*BBMN68_1709*	90.1	204.51	2.27*	M	COG1215		Glycosyltransferase for cell wall membrane
*BBMN68_1494*	81.69	173.01	2.12*	M	COG0463	*wcaA4*	Glycosyltransferase for cell wall membrane
*BBMN68_851*	60.38	123.92	2.05*	M	COG0438	*rfaG2*	Glycosyltransferase
*BBMN68_1010*	155.49	576.65	3.71*	D	COG0489		Etk-like tyrosine kinase involved in EPS biosynthesis

* changed by more than 2 fold.

**Figure 5:**
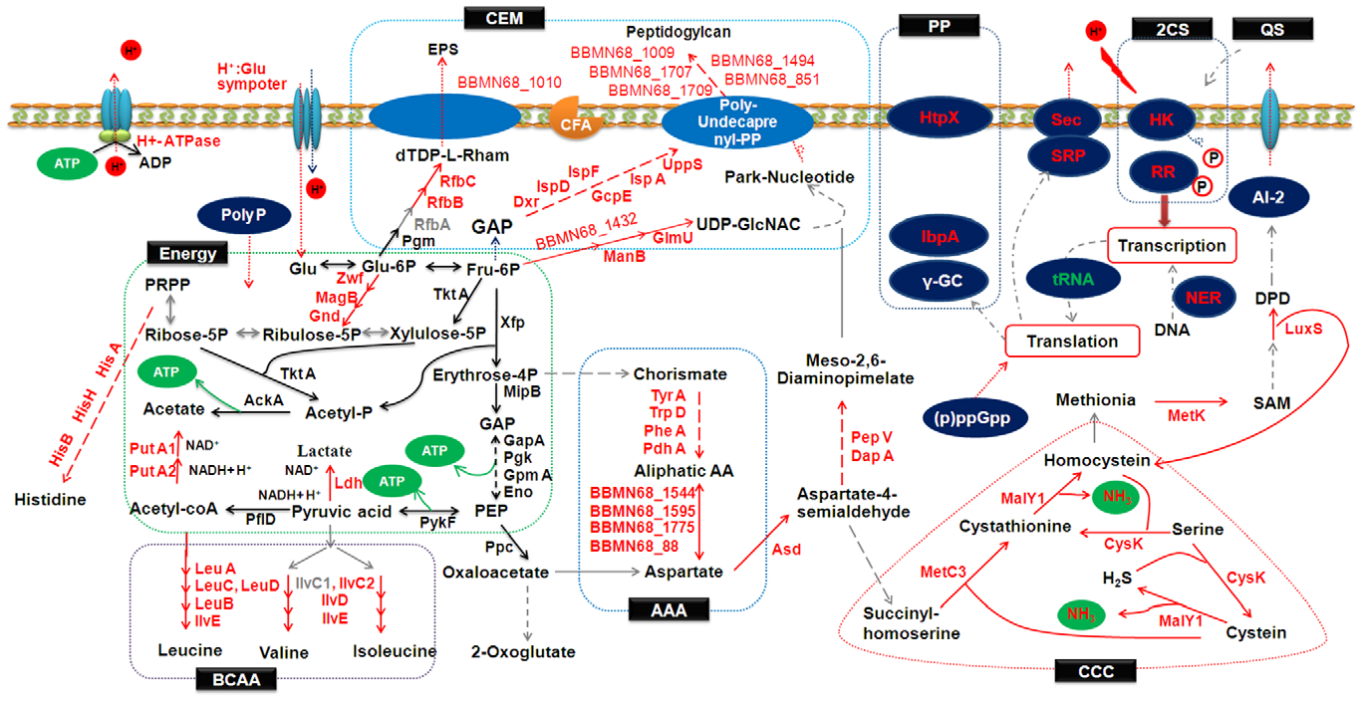
Metabolic net work of the mechanism involved in ATR in *Bifidobacterium longum* subsp. *longum* BBMN68. CEM: cell envelope and membrane; CCC: cysteine and cystathionine cycle; BCAA: branched-chain amino acids; AAA: aliphatic amino acids; PP: protein protection; 2CS: two-component system (HK: sensor; RR: response regulator); QS: quorum sensing; CFA: cyclopropane fatty acid; EPS: extracellular polysaccharide; SAM: S-adenosylmethionine.

In this study, three pieces of evidence indicated that peptidoglycan synthesis had been induced in acid-adapted cells. As shown in Table 2 and Figure 5, first, the genes (*BBMN68_1432*, *BBMN68_949*, *BBMN68_725*, *BBMN68_956* and *BBMN68_615*) involved in the synthesis of the two main precursors of peptidoglycan, UDP-acetyl-glucosamine and meso-2, 6-diaminopimelate, were upregulated by more than 2 fold. Second, poly-undecaprenyl-PP (UND-PP), which is a lipid carrier involved in polysaccharide and peptidogylcan assembly, carried glycogen to pass the membrane [32]. In the biosynthetic pathway of UND-PP, the genes encoding Dxr, IspD, IspE, GcpE, IspA, and UppS were upregulated by more 2 fold (Table 2). Third, several genes encoding glycosyltransferase involved in helping peptidoglycan to form a network by transglycosylation were upregulated by more than 2 fold (Table 2). All these results suggested that the synthesis of peptidoglycan was induced after acid-adaptation to improve the acid resistance of BBMN68.

In addition, in our study, several genes involved in the synthesis of EPS were found to have been upregulated by more than 2 fold after acid-adaptation, as shown in Figure 5 and Table 2.

These results indicated that the synthesis of CFA, EPS, and peptidoglycan constituting the cell envelope and membrane were induced in the acid-adapted cells. It was inferred that strengthening the intensity of cell wall and changing the composition of FA to block H^+^ was a strategy of ATR in BBMN68.

### Discharging H^+^

**Figure 6:**
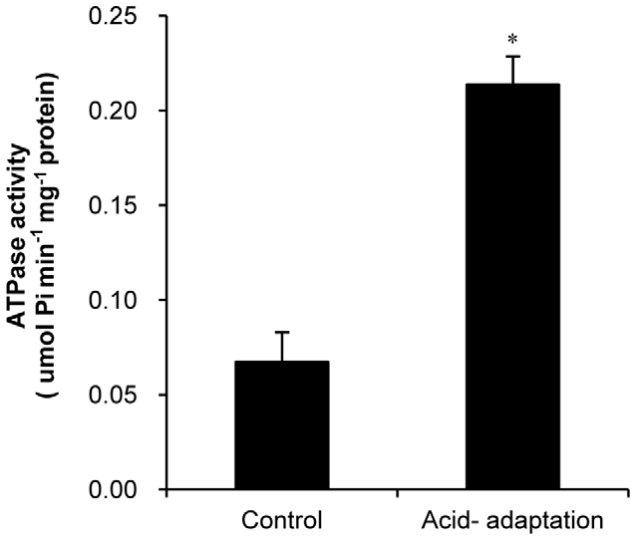
Activity of ATPase in *Bifidobacterium longum* subsp. *longum* BBMN68. * significant difference (*P*<0.05).

Generally, when bacteria are exposed to acidic conditions, pH homeostasis is maintained by discharging H^+^ by H^+^- ATPase [5], [15]. As shown in Table 3, Genes *BBMN68_1117-1124*, which together encode the 8 subunits of F_0_F_1_-ATPase in BBMN68, were upregulated after acid-adaptation. Of these, four genes were upregulated by more than 2 fold. These results implied that the activity of F_0_F_1_-ATPase may increase in ATR. To confirm this, the ATPase activity of acid-adapted BBMN68 was detected (Figure 6). After acid-adaptation at pH 4.5 lasting 2 hours, ATPase activity was found to be 3 times that of the control cells. In this way, F_0_F_1_-ATPase played an indispensable role in the ATR of BBMN68.

**Table 3:** Expression of genes encoding subunits of F_0_F_1_-ATPase in BBMN68.

Gene ID	RPKM-control	RPKM-acid-adapted	Fold change	COG	Code	Gene	Gene description
*BBMN68_1117*	3937.05	4499.09	1.14	C	COG0355	*atpC*	K02114, F-type H^+^-transporting ATPase subunit epsilon [EC:3.6.3.14]
*BBMN68_1118*	4118.78	6059.24	1.47	C	COG0055	*atpD*	K02112, F-type H^+^-transporting ATPase subunit beta [EC:3.6.3.14]
*BBMN68_1119*	2955.1	6406.36	2.17*	C	COG0224	*atpG*	K02115, F-type H^+^-transporting ATPase subunit gamma [EC:3.6.3.14]
*BBMN68_1120*	3296.97	5946.07	1.80	C	COG0056	*atpA*	K02111, F-type H^+^-transporting ATPase subunit alpha [EC:3.6.3.14]
*BBMN68_1121*	3531.07	9305.03	2.64*	C	COG0712	*atpH*	K02113, F-type H^+^-transporting ATPase subunit delta [EC:3.6.3.14]
*BBMN68_1122*	2110.9	3318.5	1.57	C	COG0711	*atpF*	K02109, F-type H^+^-transporting ATPase subunit b [EC:3.6.3.14]
*BBMN68_1123*	1993.61	4012	2.01*	C	COG0636	*atpE*	K02110, F-type H^+^-transporting ATPase subunit c [EC:3.6.3.14]
*BBMN68_1124*	599.22	2135.16	3.56*	C	COG0356	*atpB*	K02108, F-type H^+^-transporting ATPase subunit a [EC:3.6.3.14]

* changed by more than 2 fold.

### Neutralizing H^+^

The genes *BBMN68_1526* encoding cystathionine beta-lyase (MalY1), *BBMN68_917* encoding cystathionine gamma-synthase (MetC3) and *BBMN68_918* encoding cystathionine beta-synthase (CysK) involved in cysteine metabolism pathway were upregulated by 9.25, 2.71, and 2.84 fold, respectively (Table 4). The reactions catalyzed by the three enzymes constituted a NH_3_-producing cycle, here called the cysteine-cystathionine-cycle (CCC). As shown in Figure 5, MalY1 catalyzes the conversion of cystathionine to homocysteine and NH_3_. Then homocysteine combined with sercine is catalyzed to produce cystathionine by CysK. Second, MalY1 catalyzes the conversion of cysteine to H_2_S and NH_3_, then H_2_S combined with sercine is catalyzed to produce cysteine by CysK. Third, cysteine combined with succinyl-homoserine is catalyzed to produce cystathionine by MetC3. The activity of enzymes involved in this cycle was upregulated, suggesting that the ability to produce NH_3_ using CCC was promoted by acid-adaptation. Previous studies have demonstrated that NH_3_ could neutralize H^+^ to enhance the acid tolerance of bacteria [13], [19], [20]. However, CCC has not been reported as a metabolic pathway to produce NH_3_ in ATR. In our study, it was found that CCC cycle participated in ATR in bifidobacteria as NH_3_ producer. This prompted us to try to improve the acid-resistance of bifidobacteria by adding cysteine and cystathionine into the acid-stress medium.

**Table 4:** Changes in expression of genes associated with amino acid metabolism pathway.

Gene ID	RPKM-control	RPKM-acid-adapted	Fold change	COG	Code	Gene	Gene description
*BBMN68_1526*	30.77	284.73	9.25*	E	COG1168	*malY1*	K14155, cystathione beta-lyase [EC:4.4.1.8]
*BBMN68_14*	297.44	845.07	2.84*	H	COG0192	*metK*	K00789, S-adenosylmethionine synthetase [EC:2.5.1.6]
*BBMN68_918*	265.56	744	2.80*	E	COG0031	*cysK*	K01697, cystathionine beta-synthase [EC:4.2.1.22]
*BBMN68_917*	359.99	976.06	2.71*	E	COG0626	*metC3*	K01739, cystathionine gamma-synthase [EC:2.5.1.48]
*BBMN68_1263*	2667.41	18881.2	7.08*	EH	COG0059	*ilvC2*	K00053, ketol-acid reductoisomerase [EC:1.1.1.86]
*BBMN68_16*	99.73	224.99	2.26*	EG	COG0129	*ilvD*	K01687, dihydroxy-acid dehydratase [EC:4.2.1.9]
*BBMN68_592*	395.39	861.86	2.18*	EH	COG0115	*ilvE*	K00826, branched-chain amino acid aminotransferase [EC:2.6.1.42]
*BBMN68_1222*	366.15	825.87	2.26*	E	COG0119	*leuA*	K01649, 2-isopropylmalate synthase [EC:2.3.3.13]
*BBMN68_984*	237.25	1102.93	4.65*	E	COG0473	*leuB*	K00052, 3-isopropylmalate dehydrogenase [EC:1.1.1.85]
*BBMN68_1521*	37.07	107.49	2.90*	E	COG0065	*leuC*	K01703, 3-isopropylmalate/(R)-2-methylmalate dehydratase large subunit [EC:4.2.1.33 4.2.1.35]
*BBMN68_1522*	84.05	216.49	2.58*	E	COG0066	*leuD*	K01704, 3-isopropylmalate/(R)-2-methylmalate dehydratase small subunit [EC:4.2.1.33 4.2.1.35]
*BBMN68_185*	187.29	448.27	2.39*	E	COG0106	*hisA*	K01814, phosphoribosylformimino-5-aminoimidazole carboxamide ribotide isomerase [EC:5.3.1.16]K01817, phosphoribosylanthranilate isomerase [EC:5.3.1.24]
*BBMN68_182*	119.3	236.83	1.99*	E	COG0131	*hisB*	K01693, imidazoleglycerol-phosphate dehydratase [EC:4.2.1.19]
*BBMN68_184*	53.57	128	2.39*	E	COG0118	*hisH*	K02501, glutamine amidotransferase [EC:2.4.2.-]
*BBMN68_274*	21.46	45.68	2.13*	E	COG0287	*pdhA*	K04517, prephenate dehydrogenase [EC:1.3.1.12]
*BBMN68_273*	28.27	61.79	2.19*	E	COG0077	*pheA*	K14170, chorismate mutase/prephenate dehydratase [EC:5.4.99.5 4.2.1.51]
*BBMN68_312*	52.76	202.97	3.85*	E	COG0547	*trpD*	K00766, anthranilate phosphoribosyltransferase [EC:2.4.2.18]
*BBMN68_1136*	35.29	115.15	3.26*	E	COG1605	*tyrA*	K04092, chorismate mutase [EC:5.4.99.5]
*BBMN68_1775*	35.39	180	5.09*	E	COG0436		Aspartate/tyrosine/aromatic aminotransferase
*BBMN68_1595*	46.59	110.62	2.37*	E	COG0436		Aspartate/tyrosine/aromatic aminotransferase
*BBMN68_88*	71	167.86	2.36*	E	COG0436		Aspartate/tyrosine/aromatic aminotransferase
*BBMN68_1544*	245	572.85	2.34*	E	COG0436		Aspartate/tyrosine/aromatic aminotransferase
*BBMN68_1747*	122.62	299.33	2.44*	E	COG0683	*livK*	K01999, branched-chain amino acid transport system substrate-binding protein
*BBMN68_1748*	194.66	173.56	−1.12	E	COG0559	*livH*	K01997, branched-chain amino acid transport system permease protein
*BBMN68_1749*	162.21	270.74	1.67	E	COG0559	*livM*	K01998, branched-chain amino acid transport system permease protein
*BBMN68_1750*	161.77	302	1.87	E	COG0411	*livG*	K01995, branched-chain amino acid transport system ATP-binding protein
*BBMN68_1751*	285.24	650.6	2.28*	E	COG0410	*livF*	K01996, branched-chain amino acid transport system ATP-binding protein

* changed by more than 2 fold.

### Protection of proteins

The excess H^+^ entering the cell can damage the structure of some proteins. The repair of acid-induced protein damage is an important response to acid stress in LABs [5], [8], [17]. DnaK, DnaJ1, DnaJ2, GrpE, Hsp60, and Hsp10 are common general stress response proteins (GSPs) involved in ATR in other bacteria. In the acid-adapted cells, the expression of the genes encoding these GSPs changed by less than 2 fold (Table 5). These results indicated that these GSPs did not make much contribution to ATR in BBMN68. This is consistent with the results of Sanchez's 2D-Gel study, which indicated that DnaJ and GroES were down-regulated in *Bifidbacterium* under acid-stress conditions [13]. However, the genes *BBMN68_1278* and *BBMN68_1305* encoding Zn-dependent protease (HtpX) and a small heat shock molecular chaperone (IbpA), were upregulated by 4.11 and 2.83 fold (Table 5). The pH challenge was found to induce denaturation and inactivation of membrane proteins. The resulting accumulation of abnormal membrane proteins was found to disturb membrane structure and function, eventually compromising cellular integrity and viability. Previous studies have indicated that HtpX might participate in quality control of membrane proteins [33]. The significantly upregulated expression of *htpX* suggested that the quality control system was initiated to monitor protein folding states, eliminates and/or repairs the damaged proteins quickly in the acid- adapted cells. IbpA is a small heat shock protein. It is a widely distributed molecular chaperone that binds to misfolded proteins to prevent irreversible aggregation, and aids in refolding denatured proteins into competent ones [34]. This information was obtained from the studies on heat stress and oxygen stress of *E. coli* and other bacteria [35]. However, there has been no report stating that *htpX* and *ibpA* were involved in ATR in LAB. Our results provide the first evidence that *htpX* and *ibpA* may participate in ATR in *Bifidobacterium*.

**Table 5:** Expression of genes encoding molecular chaperones in BBMN68.

Gene ID	RPKM-control	RPKM-acid-adapted	Fold change	COG	Code	Gene	Gene description
*BBMN68_410*	93.5	164.44	1.76	O	COG0484	*dnaJ1*	K03686, molecular chaperone DnaJ
*BBMN68_1250*	195.29	189.37	−1.03	O	COG2214	*dnaJ2*	K03686, molecular chaperone DnaJ
*BBMN68_1251*	385.11	310.71	−1.23	O	COG0576	*grpE*	K03687, molecular chaperone GrpE
*BBMN68_1252*	845.41	773.5	−1.09	O	COG0443	*dnaK*	K04043, molecular chaperone DnaK
*BBMN68_1278*	182.36	764.67	4.19*	O	COG0501	*htpX*	K03799, heat shock protein HtpX [EC:3.4.24.-]
*BBMN68_1305*	520.3	1506.83	2.90*	O	COG0071	*ibpA*	Heat shock molecular chaperone
*BBMN68_1589*	439.28	700.76	1.60	O	COG0234	*groS*	K04078, chaperonin GroES, HSP10
*BBMN68_44*	724.67	1248.64	1.72	O	COG0459	*groL*	K04077, chaperonin GroEL, HSP60
*BBMN68_52*	474.75	659.97	1.39	O	COG0542	*clpA1*	K03696, ATP-dependent Clp protease ATP-binding subunit ClpC
*BBMN68_1510*	169.21	142.65	−1.19	O	COG0542	*clpA2*	K03695, ATP-dependent Clp protease ATP-binding subunit ClpB
*BBMN68_691*	110.28	149.64	1.36	O	COG1219	*clpX*	K03544, ATP-dependent Clp protease ATP-binding subunit ClpX
*BBMN68_692*	332.15	817.73	2.46*	NO	COG0740	*clpP1*	K01358, ATP-dependent Clp protease, protease subunit [EC:3.4.21.92]
*BBMN68_693*	524.61	949.8	1.81	NO	COG0740	*clpP2*	K01358, ATP-dependent Clp protease, protease subunit [EC:3.4.21.92]
*BBMN68_1790*	69.85	216.21	3.10*	H	COG3572	*gshA*	K01919, glutamate–cysteine ligase [EC:6.3.2.2]

* changed by more than 2 fold.

The members of the Clp protease family are involved in heat- and osmotic- tolerance in *Bifidobacterium* [36], [37]. In this study, it was found that *BBMN68_692* encoding protease subunit of Clp family was upregulated by 2.46 fold in ATR (Table 5). Such result suggested that the roles of Clp family in *Bifidobacterium* in response to the stress enviroments might be further enriched.

Low-molecular-mass thiols, such as glutathione (GSH) have been shown to possess the ability to strengthen the acid resistance of the LAB by protecting some proteins from damage. This is because they can form mixed disulfides with protein SH groups [38], [39]. GSH is synthesized from glutamate through two enzymatic processes: First, γ-glutamylcysteine (γ-GC) synthetase (GshA) ligates L-cysteine to L-glutamate at the expense of ATP, forming γ-GC. Second, GSH synthetase (GshB) adds glycine to γ-GC to form the tripeptide GSH. Kim has reported that γ-GC acts as major low-molecular-mass thiols instead of GSH in some LABs in which *gshB* is not detectable [39]. In this study, no gene homologous to *gshB* was found in the BBMN68 genome, the *BBMN68_1790* encoding GshA was upregulated by 3.1 fold (Table 5). This implied that γ-GC might contribute to the protection of protein. This suggested that the acid resistance of bifidobacteria could be improved by adding γ-GC to the acid-stress medium.

### Repair of damaged DNA

The excess H^+^ entering the cell also threatened the correctness of DNA replication and transcription by depurination and depyrimidination of DNA. To combat the acid stress, DNA repair systems were initiated in the LAB [16], [18]. The SOS and nucleotide excision repair (NER, which includes the NER-UVR and NER-Vsr systems) are the major DNA repair systems in bacteria. The changes in the expression of the genes involved in DNA repair system are shown in Table 6.

**Table 6:** Expression of genes encoding proteins involved in DNA repair in BBMN68.

Gene ID	RPKM-control	RPKM-acid-adapted	Fold change	COG	Code	Gene	Gene description
*BBMN68_394*	131.73	185.06	1.40	L	COG0178	*uvrA1*	K03701, excinuclease ABC subunit A
*BBMN68_833*	47.46	73.24	1.54	L	COG0178	*uvrA2*	K03701, excinuclease ABC subunit A
*BBMN68_740*	29.91	77.39	2.59*	L	COG0556	*uvrB*	K03702, excinuclease ABC subunit B
*BBMN68_395*	120.44	114.82	−1.05	L	COG0322	*uvrC*	K03703, excinuclease ABC subunit C
*BBMN68_138*	17.02	41.6	2.44*	L	COG0210	*uvrD1*	K03657, ATP-dependent DNA helicase II PcrA [EC:3.6.4.12]
*BBMN68_630*	58.46	99.48	1.70	L	COG0210	*uvrD2*	K03657, ATP-dependent DNA helicase II PcrA [EC:3.6.4.12]
*BBMN68_959*	59.77	96.17	1.61	L	COG0210	*uvrD3*	K03657, ATP-dependent DNA helicase II PcrA [EC:3.6.4.12]
*BBMN68_960*	87.56	50.69	−1.72	L	COG0210	*uvrD4*	Superfamily I DNA and RNA helicase
*BBMN68_93*	57.73	70.27	1.22	L	COG0272	*lig*	K01972, DNA ligase (NAD^+^) [EC:6.5.1.2]
*BBMN68_735*	163.86	248.39	1.52	L	COG0749	*polA*	K02335, DNA polymerase I [EC:2.7.7.7]
*BBMN68_773*	150.39	153.2	1.02	LK	COG1197	*mfd*	K03723, transcription-repair coupling factor [EC:3.6.4.-]
*BBMN68_1773*	40.96	112.16	2.74*			*vsr*	K07458, DNA mismatch endonuclease, patch repair protein [EC:3.1.-.-]
*BBMN68_195*	2633.17	415.81	−6.33*	KT	COG1974	*lexA1*	K01356, SOS-response transcriptional repressor LexA [EC:3.4.21.88]
*BBMN68_752*	249.42	206.3	−1.20	KT	COG1974	*lexA2*	SOS-response transcriptional repressor
*BBMN68_305*	350.71	240.03	−1.47	L	COG0468	*recA*	K03553, recombination protein RecA
*BBMN68_1092*	93.79	71.67	−1.32	LK	COG1200	*recG*	K03655, ATP-dependent DNA helicase RecG [EC:3.6.4.12]
*BBMN68_1369*	121	141.8	1.17	L	COG1195	*recF*	K03629, DNA replication and repair protein RecF
*BBMN68_1232*	74.69	99.3	1.33	L	COG0353	*recR*	K06187, recombination protein RecR
*BBMN68_146*	46.76	79.7	1.70	L	COG1381	*recO*	K03584, DNA repair protein RecO (recombination protein O)

* changed by more than 2 fold.

LexA, a member of the SOS system, represses the expression of some response genes including *lexA* itself. When the SOS system is started, the repressional activity of LexA becomes inactivated, and then the expression of the response genes is induced. In this study, the expression of *BBMN68_195* encoding LexA was downregulated by 6.33 fold in ATR (Table 6). This may have been the result of the activity of the LexA repressor, which was not removed in the acid adapted cells. These results implied that SOS was not involved in ATR in BBMN68.

In the NER-UVR system, the UVR family proteins repair the damaged DNA cooperating with DNA ligase, and DNA polymerase I. In this study, after acid adapation, the expression of *uvrB* and *uvrD* was upregulated by more than 2 fold (Table 6). This shift to upregulation implied that NER system was initiated in ATR in BBMN68. Moreover, *BBMN68_1773* encoding short patch repair endonuclease (Vsr) belonging to NER-Vsr system was upregulated by 2.74 folds after acid-adaptation (Table 6). The result offered another evidence that NER was started in the acid-adapted BBMN68. The gene *vsr* has not been reported to be involved in the ATR in LABs in previous study.

### Regulation of signal systems and global response

In this study, RNA-Seq data analysis suggested that the expression of genes involved in global response, such as (p)ppGpp synthesis, polyP synthesis, and protein export, and genes involved in signal communication, such as two-component systems (2CS) and quorum-sensing (QS) changed significantly after acid-adaptation.

*BBMN68_331* encoding GTP pyrophosphokinase (SpoT) was upregulated by 2.05 folds (Table 7). SpoT is key to (p)ppGpp production. In response to various unfavorable growth conditions, such as a global regulators, (p)ppGpp downregulates the cellular processes needed for growth and upregulates the processes needed for survival, bringing the cells into a slow-growing state [40], [41], [42].

**Table 7:** Changes in expression of genes involved in signal transduction and global response.

Gene ID	RPKM-control	RPKM-acid-adapted	Fold change	COG	Code	Gene	Gene description
*BBMN68_914*	325.82	807.55	2.48*	T	COG1854	*luxS*	K07173, S-ribosylhomocysteine lyase [EC:4.4.1.21]
*BBMN68_1079*	16.3	49.94	3.06*	TK	COG0745	*regX3*	K07776, two-component system, OmpR family, response regulator RegX3
*BBMN68_1080*	17.94	62.35	3.48*	T	COG0642	*vicK*	K07768, two-component system, OmpR family, sensor histidine kinase SenX3 [EC:2.7.13.3]
*BBMN68_47*	263.56	1086.19	4.12*	TK	COG0745		K02483, two-component system, OmpR family, response regulator
*BBMN68_48*	293.97	909.09	3.09*	T	COG0642	*baeS1*	K02484, two-component system, OmpR family, sensor kinase [EC:2.7.13.3]
*BBMN68_1518*	305.06	816.15	2.68*	P	COG0855	*ppk*	K00937, polyphosphate kinase [EC:2.7.4.1]
*BBMN68_331*	285.11	596.6	2.09*	TK	COG0317	*spoT*	K00951, GTP pyrophosphokinase [EC:2.7.6.5]
*BBMN68_1066*	97.29	209.12	2.15*	N	COG0541	*ffh*	K03106, signal recognition particle subunit SRP54
*BBMN68_1545*	296.7	1140.94	3.85*	N	COG0690	*secE*	K03073, preprotein translocase subunit SecE
*BBMN68_1285*	158.96	45.97	−3.46*	N	COG0805	*tatC*	K03118, sec-independent protein translocase protein TatC
*BBMN68_1286*	58.85	36.32	−1.62*	N	COG1826	*tatB*	K03117, sec-independent protein translocase protein TatB
*BBMN68_1284*	665.3	194.9	−3.41*	N	COG1826	*tatA*	K03116, sec-independent protein translocase protein TatA

* changed by more than 2 fold.

**Figure 7:**
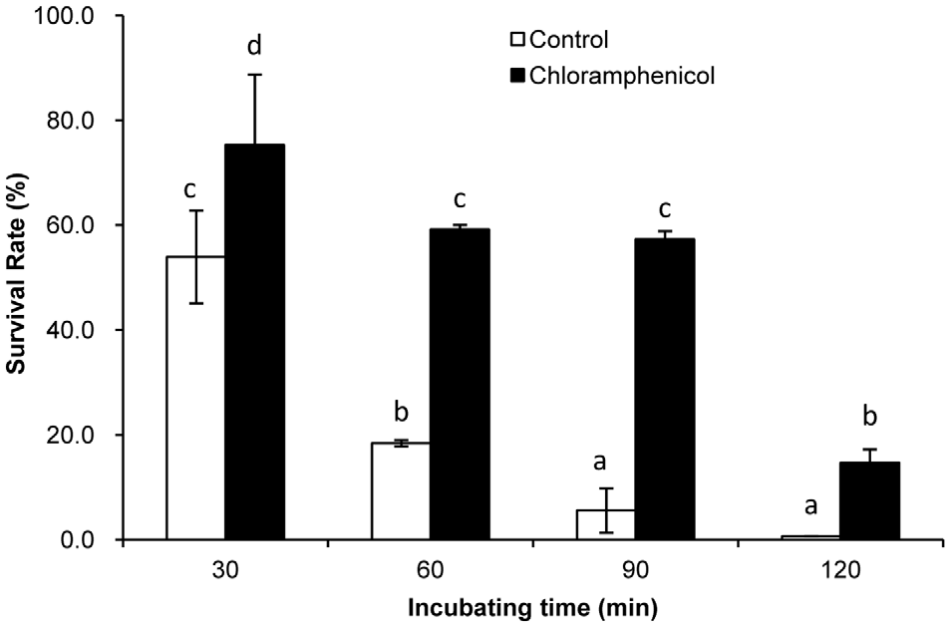
Survival rate of *Bifidobacterium longum* subsp. *longum* BBMN68 at pH 3.5 for different duration. Control cells were untreated (white block). Other cells were treated with 100 µg/ml chloramphenicol for 30 min (black block). Significant differences among the groups are marked with different letters (*P*<0.05).

The upregulated expression of *spoT* showed that acid-adaptation induced the production of (p)ppGpp to strengthen the resistance to acid challenge. Moreover, 35 of 57 genes encoding tRNA were downregulated by more than 2 fold (Table S1). This implied that the synthesis of some proteins was inhibited by the upregulation of (p)ppGpp and by the downregulation of tRNA expression in ATR in BBMN68. To determine the relationship between the inhibition of protein synthesis and acid resistance in BBMN68, the acid resistance of BBMN68 treated by chloramphenicol was assessed. Chloramphenicol was found to inhibit protein synthesis. The survival rate of BBMN68 treated with chloramphenicol remained at 57% after challenge at pH 3.5 lasting 90 min. It then decreased to 14% after challenge lasting 120 min. However, these survival rates were significantly higher than those of control cells (Figure 7). This indicated that the acid resistance of BBMN68 was strengthened when its protein synthesis was inhibited by chloramphenicol. This implied that the inhibition of protein production effected by increased production of (p)ppGpp and by reduced production of tRNA played an important role in ATR in BBMN68.

*BBMN68_1518* encoding polyphosphate kinase (PPK1) was upregulated by 2.68 fold after acid-adaptation (Table 7). PPK1 is responsible for the synthesis of Poly P, which has been shown to be essential to growth by responding to the stresses and stringencies in several bacteria. Previous studies have indicated that mutants lacking *ppk1* were defective in motility, QS and biofilm formation in *E. coli* [43], [44]. Qian has reported that oxidative stress induced the expression of *ppk* and the production of poly P in bifidobacteria, and strengthened the resistance of the cells to acid challenge [45]. For the first time, this study detected that acid-adaptation induced the expression of *ppk1* in the absence of oxidative stress.

There are two main protein export systems in the BBMN68 genome. One of them is the twin arginine targeting (TAT) system. In the BBMN68 genome, *BBMN68_1284*, *BBMN68_1285*, and *BBMN68_1286* constituted the TAT system. In the present study, these three genes were all downregulated by more than 2 fold (Table 7). These results suggested that TAT was partially shut down in ATR in BBMN68. Another system is Sec-signal recognition particle (Sec-SRP) system. *BBMN68_1545* encoding preprotein translocase subunit (SecE) and *BBMN68_1066* encoding signal recognition particle subunit SRP54 (Ffh) belonging to the Sec-SRP were upregulated by 3.85 and 2.15 fold, respectively (Table 7). Previous studies on the acid tolerance of *S. mutans* have reported that the *ffh* defective mutans is more acid-sensitive and that Ffh played an important role in several physiology processes, including pH homeostasis, pyruvate dissimilation, and sugar transport [46], [47]. *BBMN68_513* encoding signal peptidase I, which played an important role in protein export, was upregulated by more than 2 fold as well. Based on these results and the investigation in *S. mutans*, it was inferred that the Sec-SRP protein export system also played an unignorable role in ATR in BBMN68.

Transcriptional regulators are involved in adapting the cells to the ever-changing outer environment by regulating the gene expression. RNA-Seq detected that 72 genes encoding transcriptional regulators were expressed on the transcriptional level, and the expression of 28 transcriptional regulators were up- or downregulated by more than 2 fold. These results implied that transcriptional regulators participated in ATR by inducing or repressing the expression of the related genes (Table S1 and Table S2).

2CS are often involved in stress adaptation in bacteria. They act as sensors of environmental change [48]. Histidine kinase (HK) is autophosphorylated by signal stimulation. Then the phospho groups are transferred to response regulator (RR). Phosphorylation activates RR and causes it to regulate gene expression, strengthening the acid-resistance of the cell. There were 4 sets of 2CS in the BBMN68 genome, 2 of which were significantly upregulated (Table 7). *BBMN68_1079* and *BBMN68_1080*, encoding SenX3-RegX3, were upregulated by 3.06 and 3.48 fold, respectively. *BBMN68_47* and *BBMN68_48*, encoding RR and HK, were upregulated by 4.12 and 3.09 fold, respectively. Although their specific functions have not yet been determined, our results indicated that some members of the 2CSs were involved in ATR in BBMN68.

QS is a tool of interspecies communication in bacteria. Facing stress, the cell itself responds to the environment. The cell communicates with other cells by QS to increase the survival rate within the community. Autoinducer (AI-2) has been proposed as a universal signal in QS [22], [49]. LuxS catalyzes the production of (4S)-4, 5-dihydroxypentan-2, 3-dione (DPD), which is precursor of the signal molecule AI-2. Previous studies have shown that the acid resistance of *Streptococcus mutans* decreases significantly when the gene *luxS* is defective [21]. In this study, *BBMN68_914* encoding LuxS was upregulated by 2.48 folds (Table 7). From this, it was inferred that QS system might be initiated in ATR to improve the acid resistance of the whole BBMN68 community.

### Energy and substrate supply

In ATR, the cells initiated several mechanisms discussed above to improve autologous acid resistance. However, the operation of these mechanisms required energy and substrates. By analyzing the expression levels of the genes in BBMN68, we found that the cells strengthened the ability of transporting and unilizing carbon source, varied the ways of producing energy and started the mode of saving energy to guarantee the operation of those mechanisms.

RNA-Seq data suggested that there are three sugar transport systems in BBMN68 under the conditions in this study. The first is the major facilitator superfamily proteins (MFS), which allow glucose to be symported with H^+^. *BBMN68_1664* encoding the D-glucose-H^+^ symporter (GlcP), one member of the putative permeases of the MFS in BBMN68, was found to have been upregulated by 2.17 fold (Table S2). The second is the ABC-transport system, which was supported by ATP. The transcriptome data indicated that there were 8 sets of working ABC-type sugar transport systems in BBMN68, of which 6 were downregulated by more than 2 fold (Table 8). As indicated by the functional classification of membrane transport proteins in TCDB Blast (TCDB; www.tcdb.org), the substrates of the downregulated ABC transport systems did not include glucose. In this way, the changes in the expression of the genes involved in ABC transport did not affect the absorption of glucose, which is the bacterium's main carbon source. In addition to the MFS and ABC transport system, there were genes (*BBMN68_1151*, *BBMN68_1152*, and *BBMN68_1665*) encoding the three components (EI, HPr, and EII) of the glucose-specific phosphoenolpyruvate-linked phosphotransferase system (PTS) in BBMN68 genome. The expression of the genes encoding the components in PTS showed different changes: EI and HPr were upregulated by more than 2 fold (Table S2), and the expression of glucose-specific-EII was downregulated by 2.5 fold (Table S1). However, the three genes were expressed at very low levels in both the control and acid-adapted cells. For this reason, it was inferred that the PTS might not make a large contribution to glucose intake under the conditions in this study. After acid-adaptation, a sugar transport system consuming no energy was promoted, while the ATP-drived systems were shut down. This alteration caused the cells to initiate an energy-saving transport mode. The saved energy could then be used to support the discharge of H^+^ by F_0_F_1_-ATPase and other energy-consuming mechanisms of ATR in BBMN68.

**Table 8:** Expression of genes encoding the components of sugar ABC-transporter.

Gene ID	RPKM-control	RPKM-acid-adapted	Fold change	COG	Code	Gene description
*BBMN68_1168*	145.91	61.94	−2.36*	G	COG0395	K02026, multiple sugar transport system permease protein
*BBMN68_1169*	98.62	30.32	−3.25*	G	COG1175	K02025, multiple sugar transport system permease protein
*BBMN68_1170*	2153.69	298.91	−7.21*	G	COG1653	K02027, multiple sugar transport system substrate-binding protein
*BBMN68_927*	50.5	9.47	−5.33*	G	COG1653	K02027, multiple sugar transport system substrate-binding protein
*BBMN68_926*	1516.6	214.09	−7.08*	G	COG1653	K02027, multiple sugar transport system substrate-binding protein
*BBMN68_931*	1301.73	108.13	−12.04*	G	COG0395	K02026, multiple sugar transport system permease protein
*BBMN68_933*	1443.31	95.75	−15.07*	G	COG1175	K02025, multiple sugar transport system permease protein
*BBMN68_1670*	15246.02	1373.46	−11.10*	G	COG1653	K02027, multiple sugar transport system substrate-binding protein
*BBMN68_1671*	904.42	246.04	−3.68*	G	COG1175	K02025, multiple sugar transport system permease protein
*BBMN68_1672*	493.45	228	−2.16*	G	COG0395	K02026, multiple sugar transport system permease protein
*BBMN68_1422*	307.21	83.16	−3.69*	G	COG1653	K10117, multiple sugar transport system substrate-binding protein
*BBMN68_1423*	340.04	96.16	−3.54*	G	COG1175	K10118, multiple sugar transport system permease protein
*BBMN68_1424*	284.17	79.61	−3.57*	G	COG0395	K10119, multiple sugar transport system permease protein
*BBMN68_1726*	1387.95	83.55	−16.61*	G	COG1879	K10546, putative multiple sugar transport system substrate-binding protein
*BBMN68_1727*	322.43	63.43	−5.08	G	COG1129	K10548, putative multiple sugar transport system ATP-binding protein
*BBMN68_1728*	294.8	71.37	−4.13*	G	COG1172	K10547, putative multiple sugar transport system permease protein
*BBMN68_335*	96.32	47.34	−2.03*	G	COG0395	Sugar permeases
*BBMN68_919*	61.44	26.66	−2.30*	R	COG1123	ATPase components of various ABC-type transport systems
*BBMN68_920*	106.98	42.14	−2.54*	R	COG1123	ATPase components of various ABC-type transport systems
*BBMN68_217*	126.78	68.48	−1.85	G	COG1653	K02027, multiple sugar transport system substrate-binding protein
*BBMN68_218*	141.02	73.47	−1.92	G	COG1175	K02025, multiple sugar transport system permease protein
*BBMN68_219*	147.02	72.27	−2.03*	G	COG0395	K02026, multiple sugar transport system permease protein
*BBMN68_1724*	54.21	114.46	2.11*	G	COG1129	MglA2, K02056, simple sugar transport system ATP-binding protein [EC:3.6.3.17]
*BBMN68_74*	6627.48	8679.7	1.31	G	COG1879	Rbsb, K02058, simple sugar transport system substrate-binding protein
*BBMN68_75*	1201.97	2157.42	1.79	G	COG1129	MglA1, K02056, simple sugar transport system ATP-binding protein [EC:3.6.3.17]
*BBMN68_76*	948.75	1674.75	1.77	G	COG1172	AraH1, K02057, simple sugar transport system permease protein
*BBMN68_77*	712.67	1286.49	1.81	G	COG1172	AraH2, K02057, simple sugar transport system permease protein
*BBMN68_280*	567.19	734.4	1.29	R	COG1123	ATPase components of various ABC-type transport systems

* changed by more than 2 fold.

The bifidus shunt (black line in energy part in Figure 5) is a classic glycolytic pathway in bifidobacteria. Although not all of the genes involved in the bifidus shunt showed significant changes in gene expression, all were expressed at high-level in acid-adapted cells (Table 9). This suggested that the bifidus shunt continuously provided sufficient substrates and energy for ATR in BBMN68. In addition, *BBMN68_1185*, *BBMN68_1187*, and *BBMN68_1188*, which are responsible for the conversion from glucose-6-phosphate to ribulose-5-phosphate, were upregulated by more than 2 fold after acid-adaptation. This shift enhanced the organism's ability to utilize sugar and improved the efficiency of energy production in BBMN68.

**Table 9:** Expression of genes encoding the enzymes in bifidus shunt pathway.

Gene ID	RPKM-control	RPKM-acid-adapted	Fold change	COG	Code	Gene	Gene description
*BBMN68_1185*	237.79	512.44	2.16*	G	COG0364	*zwf*	K00036, glucose-6-phosphate 1-dehydrogenase [EC:1.1.1.49]
*BBMN68_1187*	80.61	173.56	2.15*	G	COG0363	*magB*	K01057, 6-phosphogluconolactonase [EC:3.1.1.31]
*BBMN68_1188*	815.25	1577.33	1.93	G	COG0362	*gnd*	K00033, 6-phosphogluconate dehydrogenase [EC:1.1.1.44]
*BBMN68_408*	1291.78	1065.78	−1.21	G	COG0021	*tkTA*	K00615, transketolase [EC:2.2.1.1]
*BBMN68_708*	15692.52	14640.71	−1.07	G	COG0021	*xfp*	K01632, fructose-6-phosphate phosphoketolase [EC:4.1.2.22]
*BBMN68_728*	1635.39	1684.62	1.03	C	COG0282	*ackA*	K00925, acetate kinase [EC:2.7.2.1]
*BBMN68_407*	5065.68	3312.2	−1.53	G	COG0176	*mipB*	K00616, transaldolase [EC:2.2.1.2]
*BBMN68_254*	4698.78	4345.62	−1.08	G	COG0057	*gapA*	K00134, glyceraldehyde 3-phosphate dehydrogenase [EC:1.2.1.12]
*BBMN68_399*	1866.21	2178.88	1.17	G	COG0126	*pgk*	K00927, phosphoglycerate kinase [EC:2.7.2.3]
*BBMN68_1687*	2517.93	3284.09	1.30	G	COG0588	*gpmA*	K01834, 2,3-bisphosphoglycerate-dependent phosphoglycerate mutase [EC:5.4.2.1]
*BBMN68_771*	2216.28	3869.75	1.75	G	COG0148	*eno*	K01689, enolase [EC:4.2.1.11]
*BBMN68_738*	2154.49	2225.71	1.03	G	COG0469	*pykF*	K00873, pyruvate kinase [EC:2.7.1.40]
*BBMN68_700*	7722.18	5888.39	−1.31	C	COG1882	*pflD*	K00656, formate C-acetyltransferase [EC:2.3.1.54]
*BBMN68_1333*	1556.45	2074.64	1.33	C	COG2352	*ppc*	K01595, phosphoenolpyruvate carboxylase [EC:4.1.1.31]
*BBMN68_193*	1305.13	3774.02	2.89*	C	COG0039	*ldh*	K00016, L-lactate dehydrogenase [EC:1.1.1.27]
*BBMN68_1612*	215.49	789.83	3.67*	C	COG1012/1454	*putA2*	K04072, acetaldehyde dehydrogenase/alcohol dehydrogenase [EC:1.2.1.10 1.1.1.1]
*BBMN68_872*	211.48	452.91	2.14*	C	COG1012	*putA1*	K00128, aldehyde dehydrogenase (NAD+) [EC:1.2.1.3]
*BBMN68_727*	40.17	102.16	2.54*	G	COG0406		K01834, 2,3-bisphosphoglycerate-dependent phosphoglycerate mutase [EC:5.4.2.1]
*BBMN68_624*	92.57	231.87	2.50*	G	COG0406		K15634, probable phosphoglycerate mutase [EC:5.4.2.1]

* changed by more than 2 fold.

In addition to energy production using carbohydrates, an energy supply pathway by utilizing branched chain amino acid (BCAA) was found in the cells after acid-adaptation. There are nine enzymes that regulate the synthetic pathway of L-leucine, L-isoleucine, and L-valine (Figure 5). The expression of the genes encoding these 9 enzymes showed an upregulated-trend. Seven of these genes were upregulated by more than 2 fold (Table 4). Previous studies have shown acid-stress-mediated metabolic shifts in leucine metabolism to produce ATP in *Lactobacillus sanfranciscensis* [50]. The shift to the biosynthetic pathway of BCAA could imply that producing ATP through leucine metabolism might meet the energy needs of the organism in BBMN68 under acid stress conditions. The shift to obtaining BCAA from the environment of the cells was also detected. *BBMN68_1747* and *BBMN68_1751*, which encode two components of ABC-type BCAA transport systems were found to have been upregulated by more than 2 fold. This provides further evidence that BCAA is essential to cells under acid stress. This suggests that the acid resistance of bifidobacteria might be improved by the addition of BCAA.

### Further discoveries

*BBMN68_182*, *BBMN68_184*, and *BBMN68_185*, encoding enzymes involved in the biosynthesis of histidine (Table 4), were upregulated by more than 2 fold. Histidine biosynthesis has been reported to be part of a stringent response in Gram-negative and Gram-positive bacteria. Broadbent has reported the intracellular accumulation of histidine in acid-adapted *Lactobacillus casei*, and adding histidine into the medium strengthened the acid tolerance at pH 2.5 [51]. This suggested that intracellular pools of histidine might also contribute to the ATR in BBMN68. Several genes in the biosynthesis pathway of aromatic amino acid (AAA) were upregulated by more than 2 folds (Table 4). *BBMN68_1775*, *BBMN68_1595*, *BBMN68_88*, and *BBMN68_1544* encoding PLP-dependent aspartate, tyrosine, and aromatic aminotransferase were differential gene expression showed (Table 4). By analyzing the metabolism pathways, it was known that aspartate metablism provided the precursors to CCC and CEM. Previous studies have shown that aspartate can strengthen acid resistance of *Lactobacillus casei* [52]. These results might be an implication that the AAA metabolism and aspartate metabolism played a role in ATR in BBMN68.

*BBMN68_14* encoding MetK was found to be upregulated by 2.84 fold after acid-adaptation. MetK was found to catalyze the synthesis of S-adenosylmethionine (SAM). SAM plays a pivotal role in promoting the stability of macromolecules under stress conditions by participating in various methylation reactions. This supplied methyl for CFA synthesis. Methyl is an essential precursor of AI-2 and other compounds [53], [54].

*BBMN68_1007* encoding 2-succinyl-5-enolpyruvyl-6-hydroxy-3-cyclohexene-1-carboxylate synthase, which is involved in the biosynthesis of vitamin K2, was found to be upregulated by 2.44 fold (Table S2, COG H).In most anaerobes and all Gram-positive aerobes, vitamin K2 is the sole electron transporter in the respiratory chain is essential to their survival [55]. *BBMN68_353* encoding Mg^2+^ transporter (CorA) was upregulated significantly by more than 2 folds (Table S2, COG P). This result was different from that of the 2010 Parry-Hanson study, which indicated that the acid-adaptation did not affect the expression of *corA* [56]. *BBMN68_1264* encoding MFS was upregulated by 16.86 fold (Table S2, COG GEPR), but the identities of its transport substrates were not clear. However, the possibility that glucose was one of the substrates that supports the organisms' energy needs could not be ignored. Also, several genes encoding transposases or putative transposases were significantly downregulated (Table S1 COG L). This trend was opposite to Broadbent's detection: the expression of the genes encoding transposase was upregulated after ATR in *Lactobacillus casei* ATCC 334 [51].

The expression of many genes was found to have changed significantly in the acid-adapted cells, however, these changes could not be explained by current theoretical findings. The expression of these genes might be regulated by RRs and by other transcriptional regulators that were stimulated by the low-pH environment. However, these alternations in gene expression reflected the physiological needs for survival of the cells in response to the environment.

The mechanism analysis of ATR in BBMN68 was summarized in Figure 5. When the cells were exposed to the low pH environment, the CEM acted as the one of first targets of high-concentration H^+^. The cell walls needed strengthening and the permeability of the membrane needed changing by alternating the fatty acid composition to keep H^+^ outside. When the H^+^ entered the cytoplasm, the cells had four main responses to the threat of H^+^: First, the F_0_F_1_-ATPase system began to discharge H^+^. Second, the ability of CCC to produce NH_3_ was strengthened to neutralize excess H^+^. Third, the cells used NER-UVR and NER-VSR to minimize the damage to DNA and used HtpX, IbpA, and γ-GC to protect proteins from acid stress. Fourth, the cells initiated global response signals ((p)ppGpp, polyP and Sec-SRP) to cause the whole cell to respond to the stress state. Besides, the cells secreted the QS signal (AI-2) to communicate among same-species cells by the cellular signal system, such as 2CS, to improve the overall survival rate. The cells also varied the pathways of producing energy by shifting to BCAA metabolism. Meanwhile, they enhanced their ability to utilize sugar to supply sufficient energy for the operation of the mechanism described above.

## Conclusion

This is the first study to analyze the mechanism behind ATR in bifidobacteria using global gene expression profiling. Differential gene expression suggests that the mechanisms of ATR in BBMN68 are involved in blocking H^+^ with CEM, discharging H^+^ with F_0_F_1_-ATPase, neutralizing H^+^ by using CCC to produce NH_3_, protecting proteins with γ-GC, HtpX and IbpA, repairing the damaged DNA with NER-UVR and NER-Vsr, regulating global response with (p)ppGpp, tRNA, ployP, and Sec-SRP, and transmitting signal using the 2CS system and QS system. Physiological testing confirmed that H^+^-ATPase played an important role in ATR, and that acid resistance could be strengthened by inhibiting protein synthesis in BBMN68. Based on this study, adding BCAA, γ-GC, cysteine, and cystathionine into the acid-stress environment may be new strategies for the improvement of acid-resistance in bifidobacteria. This information can lead to better quality control and functionality of bifidobacterial probiotic products.

## Supporting Information

Table S1 Downregulated expression genes with no detail comments in text.
(DOCX)

Table S2 Upregulated expression genes with no detail comments in text.
(DOCX)

Table S3 Summary of RNA-seq data.
(DOCX)
