# Supplementary material for: Mechanism Analysis of Acid Tolerance Response of Bifidobacterium longum subsp. longum BBMN 68 by Gene Expression Profile Using RNA-Sequencing
Source: PLoS One. 2012 Dec 7;7(12):e50777. doi: 10.1371/journal.pone.0050777 (PMC3517610; doi:10.1371/journal.pone.0050777)
Supplement: Table S1 — Downregulated expression genes with no detail comments in text. (DOCX) [file pone.0050777.s001.docx]

**Table S1. Downregulated expression genes with no detail comments in text**

| Gene ID | RPKM-  Control# | RPKM-  Acid-adaptated | Fold change | COG | Code | Gene | Gene description |
| --- | --- | --- | --- | --- | --- | --- | --- |
| *BBMN68_1706* | 4720.95 | 2376.73 | -1.99 | C | [COG1454](http://www.ncbi.nlm.nih.gov/COG/old/palox.cgi?AF0024) | *dhaT* | K00048, lactaldehyde reductase [EC:1.1.1.77] |
| *BBMN68_179* | 52.87 | 16.59 | -3.19 | C | [COG1254](http://www.ncbi.nlm.nih.gov/COG/old/palox.cgi?AF0818) | *acyP* | K01512, acylphosphatase [EC:3.6.1.7] |
| *BBMN68_1660* | 1747.03 | 244.98 | -7.13 | C | COG1249 | *lpd2* |  |
| *BBMN68_596* | 588.11 | 276.48 | -2.13 | C | [COG3288](http://www.ncbi.nlm.nih.gov/COG/old/palox.cgi?slr1239) | *pntA1* | K00324, NAD(P) transhydrogenase subunit alpha [EC:1.6.1.2] |
| *BBMN68_597* | 420.38 | 160.61 | -2.62 | C | [COG3288](http://www.ncbi.nlm.nih.gov/COG/old/palox.cgi?slr1239) | *pntA2* | K00324, NAD(P) transhydrogenase subunit alpha [EC:1.6.1.2] |
| *BBMN68_879* | 101.6 | 44.95 | -2.26 | CR | [COG0604](http://www.ncbi.nlm.nih.gov/COG/old/palox.cgi?BS_yogA) | *qor2* |  |
| *BBMN68_94* | 345.08 | 130.46 | -2.65 | D | [COG0489](http://www.ncbi.nlm.nih.gov/COG/old/palox.cgi?AF2269) | *mrp1* | K03593, ATP-binding protein involved in chromosome partitioning |
| *BBMN68_62* | 381.89 | 118.74 | -3.22 | E | [COG0834](http://www.ncbi.nlm.nih.gov/COG/old/palox.cgi?AF0231) | *hisJ1* | K10005, glutamate transport system substrate-binding protein |
| *BBMN68_63* | 278.12 | 131.49 | -2.12 | E | [COG0765](http://www.ncbi.nlm.nih.gov/COG/old/palox.cgi?AF0232) | *hisM1* | K10006, glutamate transport system permease protein |
| *BBMN68_61* | 456.37 | 133.1 | -3.43 | E | COG1126 | *hisP1* | K10008, glutamate transport system ATP-binding protein [EC:3.6.3.-] |
| *BBMN68_575* | 423.08 | 63.76 | -6.64 | E | COG0289 |  | K00215, dihydrodipicolinate reductase [EC:1.3.1.26] |
| *BBMN68_782* | 509.08 | 252.97 | -2.01 | E | [COG0498](http://www.ncbi.nlm.nih.gov/COG/old/palox.cgi?AF1316) | *ThrC* | K01733, threonine synthase [EC:4.2.3.1] |
| *BBMN68_903* | 365.61 | 184.2 | -1.98 | EP | [COG0747](http://www.ncbi.nlm.nih.gov/COG/old/palox.cgi?AF1767) |  | K02035, peptide/nickel transport system substrate-binding protein |
| *BBMN68_923* | 1494.81 | 347.5 | -4.30 | EP | COG0747 | *ddpA2* | K02035, peptide/nickel transport system substrate-binding protein |
| *BBMN68_921* | 89.41 | 36.53 | -2.45 | EP | [COG1173](http://www.ncbi.nlm.nih.gov/COG/old/palox.cgi?AF1769) | *dppC3* | K02034, peptide/nickel transport system permease protein |
| *BBMN68_901* | 36.65 | 16.92 | -2.17 | EQ | [COG3191](http://www.ncbi.nlm.nih.gov/COG/old/palox.cgi?DR0782) |  | K01266, D-aminopeptidase [EC:3.4.11.19] |
| *BBMN68_531* | 1135.05 | 490.26 | -2.32 | F | [COG0284](http://www.ncbi.nlm.nih.gov/COG/old/palox.cgi?AF0929) | *pyrF2* | K01591, orotidine-5'-phosphate decarboxylase [EC:4.1.1.23] |
| *BBMN68_1399* | 451.55 | 189.02 | -2.39 | F | [COG0209](http://www.ncbi.nlm.nih.gov/COG/old/palox.cgi?AF1664) | *nrdA* | K00525, ribonucleoside-diphosphate reductase alpha chain [EC:1.17.4.1] |
| *BBMN68_53* | 59.72 | 23.89 | -2.50 | FR | [COG0402](http://www.ncbi.nlm.nih.gov/COG/old/palox.cgi?AF0550) |  | K01485, cytosine deaminase [EC:3.5.4.1] |
| *BBMN68_234* | 469.92 | 225.45 | -2.08 | G | [COG0363](http://www.ncbi.nlm.nih.gov/COG/old/palox.cgi?BS_nagB) | *nagB* | K02564, glucosamine-6-phosphate deaminase [EC:3.5.99.6] |
| *BBMN68_1646* | 348.87 | 62.12 | -5.62 | G | COG3534 | *abfA5* | K01209, alpha-N-arabinofuranosidase [EC:3.2.1.55] |
| *BBMN68_930* | 226.02 | 72.16 | -3.13 | G | [COG1874](http://www.ncbi.nlm.nih.gov/COG/old/palox.cgi?BS_lacA) | *lacA1* | K12308, beta-galactosidase [EC:3.2.1.23] |
| *BBMN68_221* | 14.77 | 6.37 | -2.32 | G | [COG1472](http://www.ncbi.nlm.nih.gov/COG/old/palox.cgi?BS_ybbD) |  | K05349, beta-glucosidase [EC:3.2.1.21] |
| *BBMN68_1793* | 41.7 | 17.73 | -2.35 | G | [COG2730](http://www.ncbi.nlm.nih.gov/COG/old/palox.cgi?BS_bglC) | *bglC* | Endoglucanase |
| *BBMN68_650* | 295.56 | 81.89 | -3.61 | G | COG0366 | *amyA* | Glycosidase |
| *BBMN68_1462* | 240.1 | 30.69 | -7.82 | G | COG2211 | *melB3* | Na+/xyloside symporter |
| *BBMN68_1726* | 1387.95 | 83.55 | -16.61 | G | COG1879 | *xylF* | K10546, putative multiple sugar transport system substrate-binding protein |
| *BBMN68_1665* | 81.32 | 32.7 | -2.49 | G | [COG2190](http://www.ncbi.nlm.nih.gov/COG/old/palox.cgi?BS_ptsG_3) | *ptsG* | K02755 PTS system, beta-glucosides-specific IIA component [EC:2.7.1.69]  K02756 PTS system, beta-glucosides-specific IIB component [EC:2.7.1.69]  K02757  PTS system, beta-glucosides-specific IIC component |
| *BBMN68_1728* | 294.8 | 71.37 | -4.13 | G | COG1172 | *xylH* | K10547, putative multiple sugar transport system permease protein |
| *BBMN68_178* | 205.35 | 90.79 | -2.26 | G | [COG1070](http://www.ncbi.nlm.nih.gov/COG/old/palox.cgi?AF1324) | *gntK1* | K00854, xylulokinase [EC:2.7.1.17] |
| *BBMN68_1742* | 74.57 | 14.58 | -5.11 | G | COG1070 | *xylB* | K00854, xylulokinase [EC:2.7.1.17] |
| *BBMN68_1208* | 442.54 | 174.72 | -2.53 | G | [COG0676](http://www.ncbi.nlm.nih.gov/COG/old/palox.cgi?slr1438) |  | K01792, glucose-6-phosphate 1-epimerase [EC:5.1.3.15] |
| *BBMN68_1024* | 5.64 | 2.42 | -2.33 | G | COG1653 |  | K02027, multiple sugar transport system substrate-binding protein |
| *BBMN68_1392* | 2.83 | 0.15 | -18.87 | G | COG1472 |  | Hypothetical protein |
| *BBMN68_1738* | 9.67 | 0.77 | -12.56 | G | COG1172 |  | Hypothetical protein |
| *BBMN68_98* | 7.45 | 2.17 | -3.43 | G | COG0395 |  | ABC-type sugar transport system permease component |
| *BBMN68_1738* | 9.67 | 0.77 | -12.56 | G | COG1172 |  | Hypothetical protein |
| *BBMN68_98* | 7.45 | 2.17 | -2.46 | G | COG0395 |  | Sugar permeases |
| *BBMN68_1024* | 5.64 | 2.42 | -3.08 | G | COG1653 |  | Sugar-binding periplasmic proteins/domains |
| *BBMN68_1392* | 2.83 | 0.15 | -2.43 | G | COG1472 |  | Beta-glucosidase-related glycosidases |
| *BBMN68_270* | 43.28 | 19.88 | -2.18 | GEPR | [COG0477](http://www.ncbi.nlm.nih.gov/COG/old/palox.cgi?AF0008) |  | K08369, MFS transporter, putative metabolite:H+ symporter |
| *BBMN68_663* | 375.91 | 153.26 | -2.45 | GEPR | [COG0477](http://www.ncbi.nlm.nih.gov/COG/old/palox.cgi?AF0008) |  | K08217, MFS transporter, DHA3 family, macrolide efflux protein |
| *BBMN68_1354* | 51.2 | 7.48 | -6.84 | H | COG0161 | *gaBT* | K15372, taurine---2-oxoglutarate transaminase [EC:2.6.1.55] |
| *BBMN68_625* | 7.94 | 3.27 | -2.43 | I | [COG1835](http://www.ncbi.nlm.nih.gov/COG/old/palox.cgi?BS_yrhL) |  | Putative acyltransferase |
| *BBMN68_1558* | 317.66 | 123.41 | -2.57 | IQ | [COG0304](http://www.ncbi.nlm.nih.gov/COG/old/palox.cgi?BS_pksF) | *faBD* | K11533, fatty acid synthase, bacteria type [EC:2.3.1.-] |
| *BBMN68_118* | 388.61 | 88.42 | -4.40 | J | COG0480 |  | Hypothetical protein |
| *BBMN68_1661* | 3051.29 | 132.12 | -23.09 | K | COG1959 |  | Putative transcriptional regulator |
| *BBMN68_197* | 1542.2 | 349.16 | -4.42 | K | COG1327 |  | K07738, transcriptional repressor NrdR |
| *BBMN68_791* | 104.04 | 51.01 | -2.04 | K | [COG1725](http://www.ncbi.nlm.nih.gov/COG/old/palox.cgi?BS_yhcF) |  | K07979, GntR family transcriptional regulator |
| *BBMN68_303* | 2368.86 | 783.9 | -3.02 | K | [COG1396](http://www.ncbi.nlm.nih.gov/COG/old/palox.cgi?BS_sinR) |  | Xre-type transcriptional regulator |
| *BBMN68_223* | 132.96 | 60.24 | -2.21 | K | [COG1609](http://www.ncbi.nlm.nih.gov/COG/old/palox.cgi?BS_degA) |  | LacI-type response repressor |
| *BBMN68_1171* | 149.31 | 67.58 | -2.21 | K | [COG1609](http://www.ncbi.nlm.nih.gov/COG/old/palox.cgi?BS_degA) |  | K02529, LacI family transcriptional regulator |
| *BBMN68_1256* | 36.89 | 14.86 | -2.48 | K | [COG1609](http://www.ncbi.nlm.nih.gov/COG/old/palox.cgi?BS_degA) |  | LacI-type response repressor |
| *BBMN68_233* | 306.93 | 87.82 | -3.49 | K | COG1940 |  | NagC-type transcriptional regulator |
| *BBMN68_1796* | 257.16 | 69.98 | -3.67 | K | COG1846 |  | MarR-type transcriptional regulator |
| *BBMN68_934* | 231.7 | 53.03 | -4.37 | K | COG1609 |  | LacI-type response repressor |
| *BBMN68_1611* | 97.53 | 16.89 | -5.77 | K | COG1940 |  | NagC-type Transcriptional regulator |
| *BBMN68_232* | 229.78 | 17.57 | -13.08 | K | COG1940 |  | NagC-type transcriptional regulator |
| *BBMN68_1355* | 30.89 | 13.28 | -2.33 | KE | [COG1167](http://www.ncbi.nlm.nih.gov/COG/old/palox.cgi?BS_ycnF) |  | Putative transcriptional regulator |
| *BBMN68_1778* | 112.57 | 38.74 | -2.91 | KR | [COG0454](http://www.ncbi.nlm.nih.gov/COG/old/palox.cgi?AF0521) |  | Acetyltransferase |
| *BBMN68_195* | 2633.17 | 415.81 | -6.33 | KT | COG1974 | *lexA1* | K01356, repressor LexA [EC:3.4.21.88] |
| *BBMN68_793* | 164.01 | 67.37 | -2.43 | L | [COG0497](http://www.ncbi.nlm.nih.gov/COG/old/palox.cgi?BS_recN) | *recN* | K03631, DNA repair protein RecN (Recombination protein N) |
| *BBMN68_908* | 198.8 | 96.77 | -2.05 | L | [COG0358](http://www.ncbi.nlm.nih.gov/COG/old/palox.cgi?AF1899) | *dnaG* | K02316, DNA primase [EC:2.7.7.-] |
| *BBMN68_620* | 779.06 | 371.79 | -2.10 | L | [COG0816](http://www.ncbi.nlm.nih.gov/COG/old/palox.cgi?BS_yrrK) |  | K07447, putative holliday junction resolvase [EC:3.1.-.-] |
| *BBMN68_19* | 120.95 | 53.27 | -2.27 | L | [COG1198](http://www.ncbi.nlm.nih.gov/COG/old/palox.cgi?BS_priA) | *priA* | K04066, primosomal protein N' (replication factor Y) (superfamily II helicase) [EC:3.6.4.-] |
| *BBMN68_897* | 66.23 | 21.87 | -3.03 | L | [COG2801](http://www.ncbi.nlm.nih.gov/COG/old/palox.cgi?BH0335) |  | Putative transposase |
| *BBMN68_2* | 60.15 | 10.47 | -5.74 | L | COG2801 |  | Putative transposase |
| *BBMN68_1349* | 5838.84 | 1958.95 | -2.98 | L | [COG0783](http://www.ncbi.nlm.nih.gov/COG/old/palox.cgi?BS_dps) | *dps* | K04047, starvation-inducible DNA-binding protein |
| *BBMN68_898* | 90.42 | 41.84 | -2.16 | L | [COG2963](http://www.ncbi.nlm.nih.gov/COG/old/palox.cgi?BH0334) |  | K07483, transposase IS3/IS911 family proTein |
| *BBMN68_3* | 16.65 | 4.57 | -3.64 | L | COG2963 |  | Transposase |
| *BBMN68_342* | 22.18 | 10.66 | -2.08 | L | [COG2826](http://www.ncbi.nlm.nih.gov/COG/old/palox.cgi?BH2524) |  | IS30 family transposase |
| *BBMN68_1281* | 26.55 | 12.55 | -2.12 | L | [COG2826](http://www.ncbi.nlm.nih.gov/COG/old/palox.cgi?BH2524) |  | Transposase, IS30 family |
| *BBMN68_1485* | 40.05 | 17.93 | -2.23 | L | [COG2826](http://www.ncbi.nlm.nih.gov/COG/old/palox.cgi?BH2524) |  | Transposase, IS30 family |
| *BBMN68_1406* | 198.08 | 88.92 | -2.23 | M | [COG3764](http://www.ncbi.nlm.nih.gov/COG/old/palox.cgi?BH3596) | *srtA4* | K07284, sortase A [EC:3.4.22.70] |
| *BBMN68_1163* | 63.32 | 20.18 | -3.14 | N | [COG0840](http://www.ncbi.nlm.nih.gov/COG/old/palox.cgi?AF1034_2) |  | / |
| *BBMN68_613* | 159.84 | 48.18 | -3.32 | N | COG0840 |  | Hypothetical protein |
| *BBMN68_1285* | 158.96 | 45.97 | -3.46 | N | COG0805 |  | K03118, sec-independent protein translocase protein TatC |
| *BBMN68_1284* | 665.3 | 194.9 | -3.41 | N | COG1826 |  | K03116, sec-independent protein translocase protein TatA |
| *BBMN68_1241* | 4.43 | 0.99 | -4.47 | N | COG0630 | *cpaF* | Type IV secretory pathway, VirB11 components, and related ATPases involved in archaeal flagella biosynthesis |
| *BBMN68_1397* | 1042.66 | 262.67 | -3.97 | O | COG0695 | *grxC2* | K06191, glutaredoxin-like protein NrdH |
| *BBMN68_130* | 544.16 | 221.83 | -2.45 | O | [COG0330](http://www.ncbi.nlm.nih.gov/COG/old/palox.cgi?AF1420) | *hflC1* | Membrane protease-like protein |
| *BBMN68_1346* | 19754.35 | 3422.65 | -5.77 | O | COG0450 | *ahpC2* | K03386, peroxiredoxin (alkyl hydroperoxide reductase subunit C) [EC:1.11.1.15] |
| *BBMN68_684* | 21.32 | 9.42 | -2.26 | O | [COG0606](http://www.ncbi.nlm.nih.gov/COG/old/palox.cgi?slr0904) | *smS1* | K07391, magnesium chelatase family protein |
| *BBMN68_1654* | 131.23 | 50.47 | -2.60 | O | [COG1066](http://www.ncbi.nlm.nih.gov/COG/old/palox.cgi?BS_sms) | *smS2* | K04485, DNA repair protein RadA/Sms |
| *BBMN68_1345* | 2014.71 | 611.07 | -3.30 | O | COG0492 | *trxB1* | K00384, thioredoxin reductase (NADPH) [EC:1.8.1.9] |
| *BBMN68_1803* | 120.02 | 56.73 | -2.12 | P | [COG1122](http://www.ncbi.nlm.nih.gov/COG/old/palox.cgi?AF0731) |  | K02006, cobalt/nickel transport system ATP-binding protein |
| *BBMN68_1802* | 204.14 | 56.71 | -3.60 | P | COG1122 |  | K02006, cobalt/nickel transport system ATP-binding protein |
| *BBMN68_1801* | 220.07 | 69.56 | -3.16 | P | [COG0619](http://www.ncbi.nlm.nih.gov/COG/old/palox.cgi?AF0730) |  | K02008, cobalt/nickel transport system permease protein |
| *BBMN68_1065* | 104.29 | 11.77 | -8.86 | P | COG0053 |  | Putative Co/Zn/Cd cation transporter |
| *BBMN68_1149* | 234.7 | 107.92 | -2.17 | P | [COG2217](http://www.ncbi.nlm.nih.gov/COG/old/palox.cgi?AF0473) | *zntA1* | K01533, Cu2+-exporting ATPase [EC:3.6.3.4] |
| *BBMN68_1288* | 417.69 | 142.98 | -2.92 | P | [COG2217](http://www.ncbi.nlm.nih.gov/COG/old/palox.cgi?AF0473) | *zntA2* | K01552, [EC:3.6.3.-] |
| *BBMN68_1413* | 425.15 | 193.06 | -2.20 | P | [COG0474](http://www.ncbi.nlm.nih.gov/COG/old/palox.cgi?BS_yloB) | *mglA4* | Cation transport ATPase |
| *BBMN68_874* | 233.06 | 89.76 | -2.60 | P | [COG0803](http://www.ncbi.nlm.nih.gov/COG/old/palox.cgi?AF1983) | *lraI2* | K02077, zinc/manganese transport system substrate-binding protein |
| *BBMN68_1797* | 344.53 | 109.3 | -3.15 | Q | [COG1132](http://www.ncbi.nlm.nih.gov/COG/old/palox.cgi?BS_yfiC) | *mdlB6* | K06147, ATP-binding cassette, subfamily B, bacterial |
| *BBMN68_1798* | 291.79 | 82.72 | -3.53 | Q | COG1132 | *mdlB7* | K06147, ATP-binding cassette, subfamily B, bacterial |
| *BBMN68_110* | 20.48 | 9.97 | -2.05 | R | [COG1136](http://www.ncbi.nlm.nih.gov/COG/old/palox.cgi?AF1018) |  | K02068, putative ABC transport system ATP-binding protein |
| *BBMN68_1172* | 1734.16 | 455.1 | -3.81 | R | COG0661 | *aarF* | K03688, ubiquinone biosynthesis protein |
| *BBMN68_135* | 179.24 | 78.02 | -2.30 | R | [COG0613](http://www.ncbi.nlm.nih.gov/COG/old/palox.cgi?AF0505) |  | K07053Putative metal-dependent phosphoesterse |
| *BBMN68_1350* | 199.47 | 83.75 | -2.38 | R | [COG1373](http://www.ncbi.nlm.nih.gov/COG/old/palox.cgi?HI1038) |  | K07133, Putative AAA+ superfamily ATPase |
| *BBMN68_1417* | 180.44 | 68.37 | -2.64 | R | [COG0400](http://www.ncbi.nlm.nih.gov/COG/old/palox.cgi?BS_yodD) |  | K06999, Hypothetical esterase |
| *BBMN68_1447* | 289.81 | 49.32 | -5.88 | R | COG1672 |  | K06921, Putative AAA+ superfamily ATPase |
| *BBMN68_1535* | 873.85 | 271.94 | -3.21 | R | [COG1136](http://www.ncbi.nlm.nih.gov/COG/old/palox.cgi?AF1018) |  | K02003, putative ABC transport system ATP-binding protein |
| *BBMN68_1555* | 60.42 | 13.95 | -4.33 | R | COG1268 | *bioY* | K03523, putative biotin biosynthesis protein BioY |
| *BBMN68_1816* | 1498.72 | 53.06 | -28.25 | R | COG1203 |  | K07012, Hypothetical protein |
| *BBMN68_350* | 10883.6 | 2138.69 | -5.09 | R | COG3576 |  | K07006, Putative flavin-nucleotide-binding protein |
| *BBMN68_64* | 191.02 | 61.08 | -3.13 | R | [COG0842](http://www.ncbi.nlm.nih.gov/COG/old/palox.cgi?AF1005) | *hisM2* | K10007, glutamate transport system permease protein |
| *BBMN68_794* | 244.5 | 120.64 | -2.03 | R | [COG0061](http://www.ncbi.nlm.nih.gov/COG/old/palox.cgi?AF2373) |  | K00858, NAD+ kinase [EC:2.7.1.23] |
| *BBMN68_881* | 438.02 | 118.98 | -3.68 | R | COG0577 |  |  |
| *BBMN68_883* | 2727.46 | 250.26 | -10.90 | R | COG1136 |  |  |
| *BBMN68_889* | 277.36 | 75.3 | -3.68 | R | COG1373 |  | K07133, Putative AAA+ superfamily ATPase |
| *BBMN68_893* | 37.01 | 13.17 | -2.81 | R | [COG1540](http://www.ncbi.nlm.nih.gov/COG/old/palox.cgi?BS_ycsF) |  | K07160, UPF0271 protein |
| *BBMN68_919* | 61.44 | 26.66 | -2.30 | R | [COG1123](http://www.ncbi.nlm.nih.gov/COG/old/palox.cgi?sll1927) | *appF3* | K02032, peptide/nickel transport system ATP-binding protein |
| *BBMN68_920* | 106.98 | 42.14 | -2.54 | R | [COG1123](http://www.ncbi.nlm.nih.gov/COG/old/palox.cgi?sll1927) | *dppD* | K02031, peptide/nickel transport system ATP-binding protein |
| *BBMN68_1148* | 355.79 | 45.42 | -7.83 | S | COG1937 | *nreA* | Hypothetical protein |
| *BBMN68_1293* | 52.58 | 19.98 | -2.63 | S | [COG0393](http://www.ncbi.nlm.nih.gov/COG/old/palox.cgi?AF0869) |  | Hypothetical protein |
| *BBMN68_1644* | 60.23 | 25.88 | -2.33 | S | [COG2308](http://www.ncbi.nlm.nih.gov/COG/old/palox.cgi?sll0335) |  | Hypothetical protein |
| *BBMN68_1758* | 70.33 | 32.61 | -2.16 | S | [COG2078](http://www.ncbi.nlm.nih.gov/COG/old/palox.cgi?AF1969) |  | Hypothetical protein |
| *BBMN68_336* | 48.54 | 22.89 | -2.12 | S | [COG3177](http://www.ncbi.nlm.nih.gov/COG/old/palox.cgi?Z5009) |  | Hypothetical protein |
| *BBMN68_666* | 1288.76 | 619.56 | -2.08 | S | [COG3304](http://www.ncbi.nlm.nih.gov/COG/old/palox.cgi?yccF) |  | Hypothetical Membrane protein |
| *BBMN68_1694* | 2399.11 | 1190.77 | -2.01 | T | [COG0589](http://www.ncbi.nlm.nih.gov/COG/old/palox.cgi?AF0125) | *uspA2* | Universal stress protein |
| *BBMN68_51* | 718.4 | 268.14 | -2.68 | T | [COG0589](http://www.ncbi.nlm.nih.gov/COG/old/palox.cgi?AF0125) | *uspA1* | Universal stress protein |
| *BBMN68_961* | 171.15 | 82.5 | -2.07 | T | [COG0515](http://www.ncbi.nlm.nih.gov/COG/old/palox.cgi?BS_yabT) |  | Serine/threonine protein kinase |
| *BBMN68_291* | 103.42 | 46.66 | -2.22 | TK | [COG2197](http://www.ncbi.nlm.nih.gov/COG/old/palox.cgi?BS_citT) |  | Putative response regulator |
| *BBMN68_887* | 6650.29 | 2709.39 | -2.45 |  |  |  | Hypothetical protein |
| *BBMN68_249* | 3285.88 | 1469.58 | -2.24 |  |  |  | Hypothetical protein |
| *BBMN68_1442* | 1466.71 | 663.74 | -2.21 |  |  |  | Hypothetical protein |
| *BBMN68_882* | 1267.33 | 247.27 | -5.13 |  |  |  | Hypothetical protein |
| *BBMN68_1202* | 1197.73 | 403.09 | -2.97 |  |  |  | Hypothetical protein |
| *BBMN68_1583* | 1194.28 | 486.01 | -2.46 |  |  |  | AprE |
| *BBMN68_368* | 1143.03 | 251.85 | -4.54 |  |  |  | Hypothetical protein |
| *BBMN68_287* | 748.24 | 178.6 | -4.19 |  |  |  | Hypothetical protein |
| *BBMN68_1456* | 720.91 | 357.78 | -2.01 |  |  |  | CrCB4 |
| *BBMN68_275* | 677.28 | 275.06 | -2.46 |  |  |  | Hypothetical protein |
| *BBMN68_1209* | 640.47 | 225.83 | -2.84 |  |  |  | Hypothetical protein |
| *BBMN68_288* | 615.04 | 168.35 | -3.65 |  |  |  | TelA |
| *BBMN68_196* | 590.08 | 76.29 | -7.73 |  |  |  | Hypothetical protein |
| *BBMN68_932* | 510.99 | 48.05 | -10.63 |  |  |  | Hypothetical protein |
| *BBMN68_1173* | 508.38 | 187.31 | -2.71 |  |  |  | Hypothetical protein |
| *BBMN68_172* | 473.73 | 64.94 | -7.29 |  |  |  | Hypothetical protein |
| *BBMN68_1416* | 463.77 | 233.23 | -1.99 |  |  |  | Hypothetical protein |
| *BBMN68_1817* | 432.39 | 149.01 | -2.90 |  |  |  | Bacterial surface protein |
| *BBMN68_1789* | 431.96 | 120.09 | -3.60 |  |  |  | Hypothetical protein |
| *BBMN68_1505* | 386.13 | 182.74 | -2.11 |  |  |  | Hypothetical protein |
| *BBMN68_760* | 338.92 | 137.51 | -2.46 |  |  |  | Hypothetical protein |
| *BBMN68_1303* | 303.79 | 139.32 | -2.18 |  |  |  | Transposase |
| *BBMN68_475* | 301.51 | 92.79 | -3.25 |  |  |  | Hypothetical protein |
| *BBMN68_1405* | 292.1 | 116.54 | -2.51 |  |  |  | Hypothetical protein |
| *BBMN68_80* | 282.34 | 41.71 | -6.77 |  |  |  | Hypothetical protein |
| *BBMN68_1271* | 252.05 | 124.58 | -2.02 |  |  |  | Serine proteinase inhibitor |
| *BBMN68_1800* | 250.18 | 81.3 | -3.08 |  |  |  | Hypothetical protein |
| *BBMN68_1454* | 235.05 | 71.18 | -3.30 |  |  |  | Hypothetical protein |
| *BBMN68_862* | 199.47 | 75.69 | -2.64 |  |  |  | Hypothetical protein |
| *BBMN68_1204* | 194.63 | 78.91 | -2.47 |  |  |  | Hypothetical protein |
| *BBMN68_499* | 155.53 | 55.62 | -2.80 |  |  |  | Hypothetical protein |
| *BBMN68_1769* | 152.67 | 55.11 | -2.77 |  |  |  | Hypothetical protein |
| *BBMN68_1457* | 151.2 | 24.18 | -6.25 |  |  |  | BglR |
| *BBMN68_1497* | 135.92 | 68.32 | -1.99 |  |  |  | Hypothetical protein |
| *BBMN68_500* | 131.61 | 23.78 | -5.53 |  |  |  | Hypothetical protein |
| *BBMN68_935* | 128.19 | 30.8 | -4.16 |  |  |  | Hypothetical protein |
| *BBMN68_220* | 122.83 | 38.26 | -3.21 |  |  |  | Hypothetical protein |
| *BBMN68_73* | 112.73 | 43.94 | -2.57 |  |  |  | Hypothetical protein |
| *BBMN68_1404* | 110 | 41.71 | -2.64 |  |  |  | Hypothetical protein |
| *BBMN68_1666* | 109.35 | 51.28 | -2.13 |  |  |  | Hypothetical protein |
| *BBMN68_498* | 106.89 | 43.5 | -2.46 |  |  |  | Hypothetical protein |
| *BBMN68_1032* | 97.92 | 47.7 | -2.05 |  |  |  | Hypothetical protein |
| *BBMN68_189* | 96.08 | 46.43 | -2.07 |  |  |  | LytE |
| *BBMN68_154* | 93 | 18.08 | -5.14 |  |  |  | Hypothetical protein |
| *BBMN68_723* | 60.61 | 28.32 | -2.14 |  |  |  | Hypothetical protein |
| *BBMN68_894* | 59.54 | 23.43 | -2.54 |  |  |  | Hypothetical protein |
| *BBMN68_34* | 56.66 | 16.06 | -3.53 |  |  |  | RelB |
| *BBMN68_1563* | 56.46 | 28.14 | -2.01 |  |  |  | Hypothetical nucleic acid-binding protein |
| *BBMN68_1662* | 42.58 | 11.76 | -3.62 |  |  |  | Integral Membrane protein |
| *BBMN68_472* | 42.45 | 19.25 | -2.21 |  |  |  | Alpha/Beta hydrolase |
| *BBMN68_892* | 41.22 | 19.5 | -2.11 |  |  |  | Hypothetical protein |
| *BBMN68_1408* | 40.02 | 16.66 | -2.40 |  |  |  | Hypothetical protein |
| *BBMN68_155* | 36.03 | 9.58 | -3.76 |  |  |  | Hypothetical protein |
| *BBMN68_1502* | 35.46 | 13.31 | -2.66 |  |  |  | Hypothetical protein |
| *BBMN68_293* | 35.01 | 14.68 | -2.38 |  |  |  | Hypothetical protein |
| *BBMN68_783* | 34.95 | 9.84 | -3.55 |  |  |  | Hypothetical protein |
| *BBMN68_1200* | 34.13 | 6.67 | -5.12 |  |  |  | Hypothetical protein |
| *BBMN68_12* | 28.19 | 12.43 | -2.27 |  |  |  | VanZ |
| *BBMN68_33* | 23.69 | 6.3 | -3.76 |  |  |  | Hypothetical protein |
| *BBMN68_1729* | 22.41 | 4.78 | -4.69 |  |  |  | Conserved Hypothetical protein |
| *BBMN68_713* | 20.7 | 3.7 | -5.59 |  |  |  | Hypothetical protein |
| *BBMN68_1562* | 18.57 | 7.49 | -2.48 |  |  |  | Hypothetical protein |
| *BBMN68_1352* | 17.92 | 3.08 | -5.82 |  |  |  | Hypothetical protein |
| *BBMN68_1498* | 17.4 | 2.19 | -7.95 |  |  |  | Hypothetical protein |
| *BBMN68_1018* | 16.31 | 7.19 | -2.27 |  |  |  | Hypothetical protein |
| *BBMN68_170* | 13.99 | 4.93 | -2.84 |  |  |  | Hypothetical protein |
| *BBMN68_507* | 10.81 | 3.85 | -2.81 |  |  |  | Hypothetical protein |
| *BBMN68_13* | 10.16 | 2.33 | -4.36 |  |  |  | Hypothetical protein |
| *BBMN68_1566* | 9.44 | 3.84 | -2.46 |  |  |  | Hypothetical protein |
| *BBMN68_337* | 8.68 | 2.82 | -3.08 |  |  |  | Hypothetical protein |
| *BBMN68_tRNA1* | 583.69 | 55.44 | -11.11 |  |  |  | tRNA-Val |
| *BBMN68_tRNA10* | 987.95 | 164.44 | -5.88 |  |  |  | tRNA-Leu |
| *BBMN68_tRNA16* | 20.75 | 1.44 | -14.29 |  |  |  | tRNA-Pro |
| *BBMN68_tRNA17* | 134.51 | 51.7 | -2.63 |  |  |  | tRNA-Leu |
| *BBMN68_tRNA18* | 164.09 | 48.68 | -3.33 |  |  |  | tRNA-Leu |
| *BBMN68_tRNA20* | 255.34 | 47.52 | -5.26 |  |  |  | tRNA-Arg |
| *BBMN68_tRNA21* | 175.91 | 36.45 | -4.76 |  |  |  | tRNA-Thr |
| *BBMN68_tRNA23* | 712.05 | 124.55 | -5.88 |  |  |  | tRNA-Thr |
| *BBMN68_tRNA28* | 492.33 | 187.93 | -2.63 |  |  |  | tRNA-Met |
| *BBMN68_tRNA29* | 3296.24 | 203.04 | -16.67 |  |  |  | tRNA-Lys |
| *BBMN68_tRNA30* | 287 | 48.88 | -5.88 |  |  |  | tRNA-Lys |
| *BBMN68_tRNA31* | 529.23 | 135.45 | -3.85 |  |  |  | tRNA-Ser |
| *BBMN68_tRNA32* | 154.46 | 13.86 | -11.11 |  |  |  | tRNA-Thr |
| *BBMN68_tRNA33* | 3413.68 | 546.29 | -6.25 |  |  |  | tRNA-Gln |
| *BBMN68_tRNA34* | 812.48 | 247.3 | -3.33 |  |  |  | tRNA-Glu |
| *BBMN68_tRNA35* | 401.03 | 57.69 | -7.14 |  |  |  | tRNA-Gly |
| *BBMN68_tRNA36* | 1413.86 | 214.03 | -6.67 |  |  |  | tRNA-Leu |
| *BBMN68_tRNA37* | 713.22 | 52.45 | -14.29 |  |  |  | tRNA-Ile |
| *BBMN68_tRNA38* | 101 | 19.75 | -5.00 |  |  |  | tRNA-Ala |
| *BBMN68_tRNA39* | 107.94 | 23.23 | -4.55 |  |  |  | tRNA-Thr |
| *BBMN68_tRNA4* | 1220.74 | 85.35 | -14.29 |  |  |  | tRNA-Gly |
| *BBMN68_tRNA40* | 184.32 | 62.01 | -2.94 |  |  |  | tRNA-Ala |
| *BBMN68_tRNA41* | 223.13 | 51.06 | -4.35 |  |  |  | tRNA-Trp |
| *BBMN68_tRNA42* | 253.24 | 36.71 | -7.14 |  |  |  | tRNA-Gly |
| *BBMN68_tRNA43* | 272.74 | 102.77 | -2.63 |  |  |  | tRNA-Tyr |
| *BBMN68_tRNA45* | 3348.15 | 295.21 | -11.11 |  |  |  | tRNA-Met |
| *BBMN68_tRNA47* | 467.09 | 203.5 | -2.27 |  |  |  | tRNA-Ser |
| *BBMN68_tRNA48* | 37.7 | 12.6 | -3.03 |  |  |  | tRNA-Ser |
| *BBMN68_tRNA5* | 1904.68 | 340.46 | -5.56 |  |  |  | tRNA-Cys |
| *BBMN68_tRNA50* | 1485.68 | 255.65 | -5.88 |  |  |  | tRNA-Glu |
| *BBMN68_tRNA54* | 125.37 | 62.93 | -2.00 |  |  |  | tRNA-Met |
| *BBMN68_tRNA55* | 205.37 | 22.78 | -9.09 |  |  |  | tRNA-Arg |
| *BBMN68_tRNA7* | 95.95 | 22.78 | -4.17 |  |  |  | tRNA-Val |
| *BBMN68_tRNA8* | 89.74 | 10.94 | -8.33 |  |  |  | tRNA-Gly |
| *BBMN68_tRNA9* | 75.8 | 14.4 | -5.26 |  |  |  | tRNA-Pro |
