# Supplementary material for: Mechanism Analysis of Acid Tolerance Response of Bifidobacterium longum subsp. longum BBMN 68 by Gene Expression Profile Using RNA-Sequencing
Source: PLoS One. 2012 Dec 7;7(12):e50777. doi: 10.1371/journal.pone.0050777 (PMC3517610; doi:10.1371/journal.pone.0050777)
Supplement: Table S2 — Upregulated expression genes with no detail comments in text. (DOCX) [file pone.0050777.s002.docx]

**Table S2. Upregulated expression genes with no detail comments in text**

| Gene ID | RPKM-  Control# | RPKM-  Acid-adaptated | Fold change | COG | Code | Gene | Gene description |  |
| --- | --- | --- | --- | --- | --- | --- | --- | --- |
| *BBMN68_1512* | 125.87 | 251.34 | 2.00 | C | [COG0584](http://www.ncbi.nlm.nih.gov/COG/old/palox.cgi?BS_glpQ) | *ugpQ* | Glycerophosphoryl diester phosphodiesterase |  |
| *BBMN68_1128* | 2414.33 | 5127.87 | 2.12 | C | COG0221 | *ppa* | K01507, inorganic pyrophosphatase [EC:3.6.1.1] |  |
| *BBMN68_402* | 75.2 | 170.64 | 2.27 | C | COG0039 |  |  |  |
| *BBMN68_1435* | 210.72 | 1006.62 | 4.78 | C | COG0778 | *nfnB2* | K00540 , [EC:1.-.-.-] |  |
| *BBMN68_772* | 91.87 | 260.26 | 2.83 | C | COG0667 | *tas1* | Putative oxidoreductase |  |
| *BBMN68_1377* | 306.13 | 1079.12 | 3.53 | D | COG1192 | *soj4* | K03496, chromosome partitioning protein |  |
| *BBMN68_1467* | 83.05 | 210.29 | 2.53 | E | COG3579 | *pePC2* | K01372, bleomycin hydrolase [EC:3.4.22.40] |  |
| *BBMN68_1227* | 129.17 | 311.18 | 2.41 | E | COG0136 | *asd* | K00133, aspartate-semialdehyde dehydrogenase [EC:1.2.1.11] |  |
| *BBMN68_637* | 11.92 | 25.93 | 2.18 | E | COG0346 |  | Putative lactoylglutathione lyase |  |
| *BBMN68_20* | 15.47 | 43.17 | 2.79 | E | COG0560 | *serB2* | K01079, phosphoserine phosphatase [EC:3.1.3.3] |  |
| *BBMN68_850* | 107.73 | 293.69 | 2.73 | E | COG2008 | *gly1* | K01620, threonine aldolase [EC:4.1.2.5] |  |
| *BBMN68_1590* | 3.59 | 8.99 | 2.50 | E | COG0626 |  | K01761, methionine-gamma |  |
| *BBMN68_1319* | 104.69 | 209.99 | 2.01 | EH | [COG0512](http://www.ncbi.nlm.nih.gov/COG/old/palox.cgi?AF1602) | *paBA* | K01664, para-aminobenzoate synthetase component II [EC:2.6.1.85] |  |
| *BBMN68_29* | 101.03 | 215.05 | 2.13 | F | COG0015 | *purB* | K01756, adenylosuccinate lyase [EC:4.3.2.2] |  |
| *BBMN68_330* | 419.14 | 1177.99 | 2.81 | F | COG0756 | *dut* | K01520, dUTP pyrophosphatase [EC:3.6.1.23] |  |
| *BBMN68_1755* | 474.25 | 1113.48 | 2.35 | F | COG0516 | *guaB* | K00088 , IMP dehydrogenase [EC:1.1.1.205] |  |
| *BBMN68_367* | 153.08 | 335.41 | 2.19 | F | COG0516 |  | K00088, IMP dehydrogenase [EC:1.1.1.205] |  |
| *BBMN68_1756* | 59.36 | 226.62 | 3.82 | F | COG1949 | *orn2* | K13288, oligoribonuclease [EC:3.1.-.-] |  |
| *BBMN68_854* | 276.61 | 680.18 | 2.46 | F | COG0152 | *purC* | K01923, phosphoribosylaminoimidazole- succinocarboxamide synthase [EC:6.3.2.6] |  |
| *BBMN68_1218* | 92.21 | 215.07 | 2.33 | F | COG0125 | *tmk* | K00943, dTMP kinase [EC:2.7.4.9] |  |
| *BBMN68_1046* | 272.64 | 616.22 | 2.26 | F | COG0127 |  | K02428, dITP/XTP pyrophosphatase [EC:3.6.1.19] |  |
| *BBMN68_415* | 214.57 | 483.72 | 2.25 | FGR | COG0537 | *hit1* | Diadenosine tetraphosphate hydrolase |  |
| *BBMN68_1259* | 117.47 | 236.51 | 2.01 | G | [COG1640](http://www.ncbi.nlm.nih.gov/COG/old/palox.cgi?sll1676) | *malQ1* | K00705, 4-alpha-glucanotransferase [EC:2.4.1.25] |  |
| *BBMN68_1479* | 1267.43 | 4116.44 | 3.25 | G | COG1129 |  | salx-type abc antimicrobial peptide transport system atpase component |  |
| *BBMN68_188* | 39.92 | 111.83 | 2.80 | G | COG2814 | *arAJ2* | Arabinose efflux permease |  |
| *BBMN68_1812* | 19.88 | 74.44 | 3.74 | G | COG3250 | *lacZ1* | K01190, beta-galactosidase [EC:3.2.1.23] |  |
| *BBMN68_777* | 37.47 | 86.05 | 2.30 | G | COG1472 | *bglX1* | K01207, beta-N-acetylhexosaminidase [EC:3.2.1.52] |  |
| *BBMN68_1277* | 97.11 | 233.55 | 2.41 | G | COG0191 | *fBa* | K01624 fructose-bisphosphate aldolase, class II [EC:4.1.2.13] |  |
| *BBMN68_251* | 876.87 | 1908.54 | 2.18 | G | COG2017 | *galM2* | Galactose mutarotase |  |
| *BBMN68_1813* | 61.52 | 233.97 | 3.80 | G | COG2211 | *melB1* |  |  |
| *BBMN68_1151* | 24.54 | 63 | 2.57 | G | COG1080 | *ptsA* | K08483, phosphotransferase system, enzyme I, PtsI [EC:2.7.3.9] |  |
| *BBMN68_1152* | 7.77 | 18.48 | 2.38 | G | COG1925 | *ptsH* | K11189, phosphocarrier protein |  |
| *BBMN68_1264* | 130.21 | 2195.87 | 16.86 | GEPR | COG0477 |  | Putative permease |  |
| *BBMN68_1344* | 163.41 | 402.87 | 2.47 | GEPR | COG0477 |  | K08177 , MFS transporter, OFA family, oxalate/formate antiporter |  |
| *BBMN68_1664* | 549.25 | 1192.71 | 2.17 | GEPR | COG0477 | *arAJ4* | Arabinose efflux permease |  |
| *BBMN68_1735* | 7.09 | 15.4 | 2.17 | GEPR | COG0477 |  | Putative permease |  |
| *BBMN68_1007* | 198.43 | 484.39 | 2.44 | H | COG1165 |  | K02551, 2-succinyl-5-enolpyruvyl-6-hydroxy-3- cyclohexene-1-carboxylate synthase [EC:2.2.1.9] |  |
| *BBMN68_1586* | 11.94 | 28.13 | 2.36 | H | COG0212 |  | K01934, 5-formyltetrahydrofolate cyclo-ligase [EC:6.3.3.2] |  |
| *BBMN68_1719* | 131.62 | 442.16 | 3.36 | H | COG0801 | *folB* | K13940, dihydroneopterin aldolase /2-amino-4- hydroxy -6- hydroxymethyldihydropteridine diphosphokinase [EC:4.1.2.25 2.7.6.3] |  |
| *BBMN68_1718* | 79.19 | 185.82 | 2.35 | H | COG0294 | *folP* | K00796, dihydropteroate synthase [EC:2.5.1.15] |  |
| *BBMN68_983* | 33.62 | 78.72 | 2.34 | H | COG0095 | *lpLA* | K03800, lipoate-protein ligase A [EC:2.7.7.63] |  |
| *BBMN68_1048* | 76.91 | 185.27 | 2.41 | H | COG1488 | *pncB* | K00763, nicotinate phosphoribosyltransferase [EC:2.4.2.11] |  |
| *BBMN68_807* | 56.08 | 112.59 | 2.01 | H | [COG2104](http://www.ncbi.nlm.nih.gov/COG/old/palox.cgi?AF0737) | *thiS* | K03154, sulfur carrier protein |  |
| *BBMN68_315* | 143.89 | 377.71 | 2.62 | I | COG0204 | *plsC1* | K00655, 1-acyl-sn-glycerol-3-phosphate acyltransferase [EC:2.3.1.51] |  |
| *BBMN68_1412* | 176.07 | 359.71 | 2.04 | I | COG0657 |  | Esterase/lipase |  |
| *BBMN68_1310* | 40.06 | 81.84 | 2.04 | I | COG2267 | *pldB* | K01048, lysophospholipase [EC:3.1.1.5] |  |
| *BBMN68_301* | 374.93 | 758.49 | 2.02 | I | [COG0558](http://www.ncbi.nlm.nih.gov/COG/old/palox.cgi?AF0263_2) | *pgsA1* | K00995, CDP-diacylglycerol--glycerol-3-phosphate 3-phosphatidyltransferase [EC:2.7.8.5] |  |
| *BBMN68_1560* | 10.41 | 22.52 | 2.16 | I | COG0736 | *acpS* | K00997, holo-[acyl-carrier protein] synthase [EC:2.7.8.7] |  |
| *BBMN68_297* | 56.42 | 135.42 | 2.40 | J | COG0621 | *miaB* | K06168, bifunctional enzyme involved in thiolation and methylation of tRNA |  |
| *BBMN68_1064* | 94.84 | 202.48 | 2.13 | J | COG0215 | *cysS* | K01883, cysteinyl-tRNA synthetase [EC:6.1.1.16] |  |
| *BBMN68_214* | 14.7 | 43.31 | 2.95 | J | COG1490 | *dtD* | K07560, D-tyrosyl-tRNA(Tyr) deacylase [EC:3.1.-.-] |  |
| *BBMN68_1511* | 13.67 | 56.44 | 4.13 | J | COG0008 | *gltX2* | K01885, glutamyl-tRNA synthetase [EC:6.1.1.17] |  |
| *BBMN68_355* | 111.21 | 287.16 | 2.58 | J | COG0495 | *leuS* | K01869, leucyl-tRNA synthetase [EC:6.1.1.4] |  |
| *BBMN68_1466* | 318.18 | 788.69 | 2.48 | J | COG0143 | *metG* | K01874, methionyl-tRNA synthetase [EC:6.1.1.10] |  |
| *BBMN68_973* | 35.03 | 163.32 | 4.66 | J | COG0219 | *cspR* | K03216, tRNA (cytidine/uridine-2'-O-)- methyltransferase [EC:2.1.1.207] |  |
| *BBMN68_1643* | 11.72 | 39.27 | 3.35 | J | COG0101 | *truA* | K06173, tRNA pseudouridine38-40 synthase [EC:5.4.99.12] |  |
| *BBMN68_1093* | 709.57 | 2276.81 | 3.21 | J | COG0227 | *rpmB* | K02902, large subunit ribosomal protein L28 |  |
| *BBMN68_1638* | 524.95 | 1083.55 | 2.06 | J | COG0257 | *rpmJ* | K02919, large subunit ribosomal protein L36 |  |
| *BBMN68_1619* | 2144.06 | 4909.49 | 2.29 | J | COG0185 | *rpsS* | K02965, small subunit ribosomal protein S19 |  |
| *BBMN68_1156* | 4109.81 | 8399.73 | 2.04 | J | COG0360 | *rpsF* | K02990, small subunit ribosomal protein S6 |  |
| *BBMN68_1047* | 200.06 | 483.21 | 2.42 | J | COG0689 | *rph* | K00989, ribonuclease PH [EC:2.7.7.56] |  |
| *BBMN68_1145* | 50.37 | 112.58 | 2.24 | J | COG0566 | *spoU3* |  |  |
| *BBMN68_605* | 58.65 | 146.58 | 2.50 | J | COG0566 | *spoU1* | K00599 ,[EC:2.1.1.-] |  |
| *BBMN68_1328* | 111.63 | 256.65 | 2.30 | J | COG0180 | *trpS* | K01867, tryptophanyl-tRNA synthetase [EC:6.1.1.2] |  |
| *BBMN68_15* | 802.83 | 1973.15 | 2.46 | K | COG1758 | *rpoZ* | K03060, DNA-directed RNA polymerase subunit omega [EC:2.7.7.6] |  |
| *BBMN68_1057* | 89.42 | 213.02 | 2.38 | K | COG0571 | *rnc* | K03685, ribonuclease III [EC:3.1.26.3] |  |
| *BBMN68_1768* | 23.71 | 53.04 | 2.24 | K | COG2378 |  | Hypothetical transcriptional regulator |  |
| *BBMN68_1378* | 181.72 | 592.82 | 3.26 | K | COG1475 |  | K03497, chromosome partitioning protein, ParB family |  |
| *BBMN68_834* | 182.05 | 465.83 | 2.56 | K | COG0640 |  | Hypothetical protein |  |
| *BBMN68_1546* | 304.94 | 937.49 | 3.07 | K | COG0250 | *nusG* | K02601, transcriptional antiterminator NusG |  |
| *BBMN68_764* | 177.54 | 514.69 | 2.90 | K | COG0782 | *greA* | K03624, transcription elongation factor GreA |  |
| *BBMN68_1099* | 24.51 | 126.56 | 5.16 | K | COG1309 |  | AcrR-type transcriptional regulator |  |
| *BBMN68_1182* | 52.99 | 155.67 | 2.94 | K | COG1309 |  | AcrR-type transcriptional regulator |  |
| *BBMN68_1449* | 10.56 | 25 | 2.37 | K | COG1309 |  | AcrR-type transcriptional regulator |  |
| *BBMN68_525* | 103.73 | 215.59 | 2.08 | K | COG0583 | *fhuR1* | fhu operon transcription regulator |  |
| *BBMN68_96* | 1.92 | 11.88 | 6.19 | K | COG1309 |  | Hypothetical protein |  |
| *BBMN68_1811* | 49.29 | 111.98 | 2.27 | K | COG1609 |  | K02529, LacI family transcriptional regulator |  |
| *BBMN68_574* | 0.22 | 0.58 | 2.64 | K | [COG1609](http://www.ncbi.nlm.nih.gov/COG/old/palox.cgi?BS_degA) |  | LacR-type transcription regulator |  |
| *BBMN68_748* | 49.96 | 131.32 | 2.63 | K | COG1329 |  | K07736, CarD family transcriptional regulator |  |
| *BBMN68_4* | 3.1 | 8.96 | 2.89 | K | COG1396 |  | Hypothetical protein |  |
| *BBMN68_96* | 1.92 | 11.88 | 6.19 | K | COG1309 |  | Hypothetical protein |  |
| *BBMN68_574* | 0.22 | 0.58 | 2.64 | K | [COG1609](http://www.ncbi.nlm.nih.gov/COG/old/palox.cgi?BS_degA) |  | LacR-type transcription regulator |  |
| *BBMN68_96* | 1.92 | 11.88 | 6.19 | K | COG1309 |  | Transcriptional regulator |  |
| *BBMN68_479* | 9.64 | 50.18 | 5.21 | KR | COG0454 |  | Acetyltransferase |  |
| *BBMN68_1217* | 27.47 | 62.61 | 2.28 | L | COG0470 | *dnaX1* | K02341,DNA polymerase III subunit delta' [EC:2.7.7.7] |  |
| *BBMN68_1371* | 239.96 | 533.8 | 2.22 | L | COG0593 | *dnaA* | K02313, chromosomal replication initiator protein |  |
| *BBMN68_1757* | 48.49 | 126.28 | 2.60 | L | COG0507 | *recD* |  |  |
| *BBMN68_359* | 56.83 | 137.41 | 2.42 | L | COG1466 | *holA* | K02340, DNA polymerase III subunit delta [EC:2.7.7.7] |  |
| *BBMN68_1097* | 5.52 | 12.36 | 2.24 | L | COG0350 | *sdA* | K00567, methylated-DNA-[protein]-cysteine S-methyltransferase [EC:2.1.1.63] |  |
| *BBMN68_1176* | 69.63 | 154.73 | 2.22 | L | COG0305 | *dnaB* | K02314, replicative DNA helicase [EC:3.6.4.12] |  |
| *BBMN68_1000* | 55.88 | 151.99 | 2.72 | LN | COG0758 |  | K04096, DNA processing protein |  |
| *BBMN68_733* | 104.68 | 218.32 | 2.09 | LR | COG0494 | *mutT1* |  |  |
| *BBMN68_1376* | 51.85 | 161.25 | 3.11 | M | COG0357 | *gidB* | K03501, 16S rRNA (guanine527-N7)-methyltransferase [EC:2.1.1.170] |  |
| *BBMN68_1085* | 383.83 | 798.5 | 2.08 | N | COG0840 |  | Hypothetical protein |  |
| *BBMN68_1300* | 55.2 | 170.59 | 3.09 | O | COG1826 |  | K12267, peptide methionine sulfoxide reductase msrA/msrB [EC:1.8.4.11 1.8.4.12] |  |
| *BBMN68_567* | 12.94 | 37.24 | 2.88 | O | [COG1826](http://www.ncbi.nlm.nih.gov/COG/old/palox.cgi?AF2056) | *ahpC1* | K03564, peroxiredoxin Q/BCP [EC:1.11.1.15] |  |
| *BBMN68_1763* | 198.79 | 403.87 | 2.03 | O |  | *pePO* | K07386, putative endopeptidase |  |
| *BBMN68_1086* | 148.62 | 306.81 | 2.06 | O | COG0229 | *pcP* | K01304, pyroglutamyl-peptidase [EC:3.4.19.3] |  |
| *BBMN68_770* | 60.44 | 166.6 | 2.76 | O | COG1225 |  | Septum formation initiator |  |
| *BBMN68_1379* | 318.89 | 683.89 | 2.14 | O | [COG3590](http://www.ncbi.nlm.nih.gov/COG/old/palox.cgi?CC3504) | *trxB2* | K00384, thioredoxin reductase (NADPH) [EC:1.8.1.9] |  |
| *BBMN68_1289* | 1427.39 | 3602.82 | 2.52 | O | COG2039 | *DegQ* | K08372, putative serine protease PepD [EC:3.4.21.-] |  |
| *BBMN68_83* | 19.92 | 47.51 | 2.39 | P | COG2919 |  | K02006, cobalt/nickel transport system ATP-binding protein |  |
| *BBMN68_353* | 233.17 | 576.16 | 2.47 | P | COG0492 | *corA* | K03284, metal ion transporter, MIT family |  |
| *BBMN68_745* | 44.89 | 92.4 | 2.06 | PH | COG0265 | *znuC* | K02074, zinc/manganese transport system ATP-binding protein |  |
| *BBMN68_1506* | 228.35 | 457.5 | 2.00 | Q | COG1122 | *mhpD* |  |  |
| *BBMN68_1006* | 135.03 | 300.55 | 2.23 | QR | COG0598 |  | Hypothetical protein |  |
| *BBMN68_261* | 188.9 | 381.2 | 2.02 | R | COG1120 |  | K02003, putative ABC transport system ATP-binding protein  K02004, putative ABC transport system permease protein |  |
| *BBMN68_1480* | 671.97 | 1869.5 | 2.78 | R | [COG0179](http://www.ncbi.nlm.nih.gov/COG/old/palox.cgi?AF0091) |  | saly-type abc antimicrobial peptide transport system permease component |  |
| *BBMN68_192* | 9.1 | 26.69 | 2.93 | R | COG1028 | *hflX* | K03665, GTP-binding protein HflX |  |
| *BBMN68_145* | 120.55 | 257.21 | 2.13 | R | [COG1136](http://www.ncbi.nlm.nih.gov/COG/old/palox.cgi?AF1018) |  | Esterase/lipase |  |
| *BBMN68_360* | 39.55 | 94.26 | 2.38 | R | COG0577 |  | K06925, UPF0079 ATP-binding protein |  |
| *BBMN68_119* | 30.86 | 129.36 | 4.19 | R | COG2262 |  | K06999 Putative esterase |  |
| *BBMN68_376* | 84.81 | 204.57 | 2.41 | R | COG1073 |  | K06941, 23S rRNA (adenine2503-C2)- methyltransferase [EC:2.1.1.192] |  |
| *BBMN68_1478* | 32.25 | 139.04 | 4.31 | R | COG0802 |  | K06950, uncharacterized protein |  |
| *BBMN68_403* | 101.9 | 320.74 | 3.15 | R | COG0400 |  | K07024 | |
| *BBMN68_568* | 59.51 | 130.59 | 2.19 | R | COG0820 |  |  |  |
| *BBMN68_707* | 18.39 | 44.38 | 2.41 | R | COG1418 |  | K07024, Putative HAD superfamily hydrolase |  |
| *BBMN68_1291* | 7.21 | 35.49 | 4.92 | R | COG0561 |  | Hypothetical protein |  |
| *BBMN68_849* | 42.64 | 213.36 | 5.00 | R | COG0561 |  | K03453, bile acid:Na+ symporter, BASS family |  |
| *BBMN68_830* | 17.84 | 36.86 | 2.07 | R | COG0561 |  | Hypothetical permease |  |
| *BBMN68_433* | 73.99 | 263.19 | 3.56 | R | COG1272 | *gph* | K01091, phosphoglycolate phosphatase [EC:3.1.3.18] |  |
| *BBMN68_1224* | 36.1 | 165.19 | 4.58 | R | COG0385 |  | Calcineurin-like phosphoesterase |  |
| *BBMN68_591* | 33.79 | 98.87 | 2.93 | R | COG0679 |  | K02428, dITP/XTP pyrophosphatase [EC:3.6.1.19] |  |
| *BBMN68_164* | 66.87 | 244.35 | 3.65 | R | COG0546 |  | tRNA-dihydrouridine synthase |  |
| *BBMN68_1515* | 14.72 | 40.85 | 2.78 | R | COG1408 |  | K07133, Putative AAA+ superfamily ATPase |  |
| *BBMN68_302* | 211.13 | 548.81 | 2.60 | R | COG1694 | *cinA* | K03743 |  |
| *BBMN68_167* | 47.29 | 236.82 | 5.01 | S | COG0042 | *tlyC1* | K02221, YggT family protein |  |
| *BBMN68_1129* | 148.72 | 499.83 | 3.36 | S | COG1373 |  | Hypothetical Membrane protein |  |
| *BBMN68_1190* | 19.52 | 46.7 | 2.39 | S | COG1546 |  | K01421, putative membrane protein |  |
| *BBMN68_1433* | 42.14 | 98.9 | 2.35 | S | COG0762 |  | K01421, putative membrane protein |  |
| *BBMN68_1434* | 74.02 | 190.86 | 2.58 | S | COG1971 |  | K01421, putative membrane protein |  |
| *BBMN68_225* | 17.07 | 39.78 | 2.33 | S | COG1511 |  | K09704, hypothetical protein |  |
| *BBMN68_726* | 133.72 | 294.08 | 2.20 | S | COG1511 |  | K09710, ribosome-associated protein |  |
| *BBMN68_829* | 4.5 | 11.72 | 2.60 | S | COG1511 |  | Hypothetical protein |  |
| *BBMN68_1301* | 95.17 | 240.08 | 2.52 | S | COG3538 |  | Hypothetical protein |  |
| *BBMN68_1585* | 66.07 | 248 | 3.75 | S | COG0799 |  | Hypothetical protein |  |
| *BBMN68_296* | 98.26 | 340.51 | 3.47 | S | [COG0599](http://www.ncbi.nlm.nih.gov/COG/old/palox.cgi?AF0348) |  | Hypothetical protein |  |
| *BBMN68_841* | 119.55 | 291.76 | 2.44 | S | COG2898 |  | Hypothetical Membrane protein |  |
| *BBMN68_842* | 46.22 | 471.5 | 10.20 | S | COG2331 |  | Hypothetical Membrane protein |  |
| *BBMN68_21* | 26.28 | 57.9 | 2.20 | S | COG2357 |  | Hypothetical Membrane protein |  |
| *BBMN68_711* | 60.2 | 134.54 | 2.23 | T | COG2246 | *arsC1* | K03741, arsenate reductase [EC:1.20.4.1] |  |
| *BBMN68_996* | 98.68 | 481.98 | 4.88 | T | COG2855 |  | K01104, protein-tyrosine phosphatase [EC:3.1.3.48] |  |
| *BBMN68_703* | 59.73 | 132.49 | 2.22 | T | COG0586 | *abgB* |  |  |
| *BBMN68_1679* | 1350.03 | 3371.76 | 2.50 |  |  |  | Hypothetical Membrane protein |  |
| *BBMN68_1680* | 741.03 | 1932.24 | 2.61 |  |  |  | Hypothetical protein |  |
| *BBMN68_202* | 491.53 | 1385.23 | 2.82 |  |  |  | Hypothetical protein |  |
| *BBMN68_1681* | 468.44 | 1016.09 | 2.17 |  |  |  | StBC |  |
| *BBMN68_1691* | 305.28 | 611.14 | 2.00 |  |  |  | Hypothetical protein |  |
| *BBMN68_329* | 288.4 | 1335.51 | 4.63 |  |  |  | Hypothetical protein |  |
| *BBMN68_1655* | 274.88 | 761.91 | 2.77 |  |  |  | Hypothetical protein |  |
| *BBMN68_428* | 261.07 | 683.17 | 2.62 |  |  | *pepdA1* | K08659, dipeptidase [EC:3.4.-.-] |  |
| *BBMN68_142* | 225.28 | 454.76 | 2.02 |  |  |  | Hypothetical protein |  |
| *BBMN68_566* | 200.35 | 647.49 | 3.23 |  |  |  | Hypothetical protein |  |
| *BBMN68_622* | 179.66 | 512.78 | 2.85 |  |  | *luxC* | Acyl-CoA reductase |  |
| *BBMN68_967* | 152.89 | 353.82 | 2.31 |  |  | *paaK* | Coenzyme F390 synthetase |  |
| *BBMN68_245* | 142.75 | 286.17 | 2.00 |  |  | */* | Hypothetical protein |  |
| *BBMN68_1390* | 141.79 | 313.89 | 2.21 |  |  |  | Putative esterase |  |
| *BBMN68_1302* | 140.39 | 373.3 | 2.66 |  |  |  | Hypothetical protein |  |
| *BBMN68_183* | 137.26 | 307.55 | 2.24 |  |  |  | Hypothetical protein |  |
| *BBMN68_1008* | 111.57 | 332.55 | 2.98 |  |  |  | Hypothetical protein |  |
| *BBMN68_1247* | 106.84 | 292.18 | 2.73 |  |  | *dsBG* | Protein-disulfide isomerase |  |
| *BBMN68_1716* | 105.71 | 248 | 2.35 |  |  |  | Hypothetical protein |  |
| *BBMN68_1130* | 104.58 | 258.89 | 2.48 |  |  |  | Hypothetical protein |  |
| *BBMN68_141* | 99.46 | 206.48 | 2.08 |  |  |  | Hypothetical protein |  |
| *BBMN68_1184* | 97.48 | 304.24 | 3.12 |  |  |  | Hypothetical protein |  |
| *BBMN68_434* | 85.52 | 326.91 | 3.82 |  |  |  | Hypothetical protein |  |
| *BBMN68_757* | 83.36 | 201.07 | 2.41 |  |  |  | K07272 rhamnosyltransferase [EC:2.4.1.-] |  |
| *BBMN68_1674* | 82.66 | 189.53 | 2.29 |  |  |  | Hypothetical protein |  |
| *BBMN68_971* | 81.84 | 180.82 | 2.21 |  |  |  | Hypothetical protein |  |
| *BBMN68_457* | 77.4 | 261.85 | 3.38 |  |  |  | Hypothetical protein |  |
| *BBMN68_1565* | 73.78 | 147.28 | 2.00 |  |  |  | Hypothetical protein |  |
| *BBMN68_1146* | 66.71 | 137.2 | 2.06 |  |  |  | Putative homoserine kinase type II |  |
| *BBMN68_314* | 61.97 | 160.83 | 2.60 |  |  | *galT2* | K00965, UDPglucose--hexose-1-phosphate uridylyltransferase [EC:2.7.7.12] |  |
| *BBMN68_730* | 60.71 | 125.3 | 2.06 |  |  |  | Hypothetical protein |  |
| *BBMN68_1777* | 58.57 | 135.99 | 2.32 |  |  |  | Hypothetical protein |  |
| *BBMN68_1493* | 57.61 | 172.59 | 3.00 |  |  | *dppX2* | Dipeptidyl aminopeptidase/acylaminoacyl-peptidase |  |
| *BBMN68_186* | 53.69 | 122.71 | 2.29 |  |  |  | Surface antigen protein |  |
| *BBMN68_478* | 50.33 | 100.5 | 2.00 |  |  |  | Hypothetical protein |  |
| *BBMN68_1246* | 48.21 | 101.5 | 2.11 |  |  | *elsH2* | Metal-dependent hydrolase |  |
| *BBMN68_1675* | 43.86 | 114.39 | 2.61 |  |  |  | K01201, glucosylceramidase [EC:3.2.1.45] |  |
| *BBMN68_913* | 43.34 | 111.7 | 2.58 |  |  |  | Hypothetical protein / |  |
| *BBMN68_1791* | 41.34 | 90.5 | 2.19 |  |  |  | Hypothetical protein |  |
| *BBMN68_671* | 37.68 | 157.07 | 4.17 |  |  |  | Hypothetical protein |  |
| *BBMN68_1060* | 35.68 | 93.6 | 2.62 |  |  |  | Hypothetical protein |  |
| *BBMN68_661* | 33.99 | 125.22 | 3.68 |  |  |  | Hypothetical protein |  |
| *BBMN68_1299* | 30 | 112.55 | 3.75 |  |  |  | Hypothetical protein |  |
| *BBMN68_1334* | 27.16 | 69.84 | 2.57 |  |  |  | Hypothetical protein |  |
| *BBMN68_413* | 26.6 | 114.34 | 4.30 |  |  |  | Hypothetical protein |  |
| *BBMN68_665* | 26.02 | 68.93 | 2.65 |  |  |  | Hypothetical protein |  |
| *BBMN68_338* | 25.64 | 80.08 | 3.12 |  |  |  | SAM-dependent methyltransferase |  |
| *BBMN68_563* | 23.92 | 69.67 | 2.91 |  |  |  | Hypothetical protein |  |
| *BBMN68_694* | 23.71 | 54.42 | 2.30 |  |  |  | Hypothetical protein |  |
| *BBMN68_326* | 22.37 | 56.82 | 2.54 |  |  |  | K00963, UTP--glucose-1-phosphate uridylyltransferase [EC:2.7.7.9] |  |
| *BBMN68_1137* | 21.62 | 113.96 | 5.27 |  |  |  | Hypothetical protein / |  |
| *BBMN68_393* | 20.48 | 43.08 | 2.10 |  |  |  | Hypothetical protein |  |
| *BBMN68_672* | 20.48 | 104.39 | 5.10 |  |  |  | Hypothetical protein |  |
| *BBMN68_1226* | 19.79 | 54.91 | 2.77 |  |  |  | Hypothetical protein |  |
| *BBMN68_272* | 18.28 | 50.98 | 2.79 |  |  |  | Hypothetical protein |  |
| *BBMN68_831* | 17.87 | 46.79 | 2.62 |  |  |  | Hypothetical protein |  |
| *BBMN68_1553* | 17.63 | 49.06 | 2.78 |  |  | *ssl2* | DNA or RNA helicase of superfamily II |  |
| *BBMN68_581* | 17.62 | 50.28 | 2.85 |  |  |  | Hypothetical protein |  |
| *BBMN68_1225* | 17.61 | 39.23 | 2.23 |  |  |  | Hypothetical protein |  |
| *BBMN68_797* | 16.57 | 67.11 | 4.05 |  |  |  | Hypothetical protein |  |
| *BBMN68_1049* | 16.56 | 45.01 | 2.72 |  |  |  | Hypothetical protein |  |
| *BBMN68_1578* | 15.75 | 39.23 | 2.49 |  |  |  | Hypothetical protein |  |
| *BBMN68_480* | 14.77 | 76.04 | 5.15 |  |  |  | Hypothetical protein |  |
| *BBMN68_1483* | 14.31 | 65.63 | 4.59 |  |  |  | Hypothetical protein |  |
| *BBMN68_1340* | 13.69 | 31.51 | 2.30 |  |  |  | Hypothetical protein |  |
| *BBMN68_1465* | 12.05 | 26.62 | 2.21 |  |  |  | Hypothetical protein |  |
| *BBMN68_351* | 10.19 | 35.68 | 3.50 |  |  |  | Hypothetical kinase |  |
| *BBMN68_521* | 8.73 | 18.55 | 2.12 |  |  |  | Hypothetical protein |  |
| *BBMN68_470* | 7.04 | 29.74 | 4.22 |  |  |  | Hypothetical protein |  |
| *BBMN68_545* | 5.56 | 14.83 | 2.67 |  |  |  | Hypothetical protein |  |
| *BBMN68_60* | 4.57 | 18.54 | 4.06 |  |  |  | Hypothetical protein |  |
| *BBMN68_447* | 4.39 | 27.28 | 6.21 |  |  |  | Hypothetical protein |  |
| *BBMN68_618* | 4 | 22.93 | 5.73 |  |  | *wecD* | Histone acetyltransferase |  |
| *BBMN68_1330* | 3.66 | 10.64 | 2.91 |  |  |  | Hypothetical protein |  |
| *BBMN68_1464* | 2.36 | 10.66 | 4.52 |  |  |  | Hypothetical protein |  |
