# Supplementary material for: Mechanism Analysis of Acid Tolerance Response of Bifidobacterium longum subsp. longum BBMN 68 by Gene Expression Profile Using RNA-Sequencing
Source: PLoS One. 2012 Dec 7;7(12):e50777. doi: 10.1371/journal.pone.0050777 (PMC3517610; doi:10.1371/journal.pone.0050777)
Supplement: Table S3 — Summary of RNA-seq data. (DOCX) [file pone.0050777.s003.docx]

**Table S3. Summary of RNA-seq data**

| Condition | Total no. of sequenced reads | Percentage of reads unique mapped | Percentage of reads multiple mapped | Percentage of reads mapped to rRNA |
| --- | --- | --- | --- | --- |
| Control cells | 41327645 | 39.38% | 46.57% | 6.35% |
| Acid-adaptated cells | 40158788 | 44.91% | 49.01% | 3.49% |
